# Heritability of behavioral traits and their genetic correlations with carcass traits in beef cattle

**DOI:** 10.1186/s12711-026-01082-5

**Published:** 2026-08-24

**Authors:** Akio Onogi, Yuki Oya, Toshio Watanabe, Atsushi Ogino, Masakazu Shinomiya, Kazuhito Kurogi

**Affiliations:** 1 Department of Life Sciences, Faculty of Agriculture, Ryukoku University, 1-5, Yokotani, Seta, Oe-cho, Otsu, Shiga 520-2194 Japan; 2 Maebashi Institute of Animal Science, Livestock Improvement Association of Japan, Inc., Maebashi, 371-0121 Japan; 3 Cattle Breeding Department, Livestock Improvement Association of Japan, Inc., Tokyo, 135-0041 Japan

## Abstract

**Background:**

Feeding and rumination behaviors in cattle are genetically associated with feed efficiency and growth traits. However, other behavioral traits such as lying, standing, and moving remain less studied. Moreover, the genetic relationships between behavioral traits and carcass traits are poorly understood in beef cattle. This study analyzed seven behavioral classes—feeding, lying, moving, standing, ruminating, ruminating while lying, and ruminating while standing—measured using wearable sensors in 1032 Japanese Black cattle and investigated their genetic correlations with carcass traits. In addition, the potential use of these behavioral traits as secondary traits to improve the prediction accuracy of carcass breeding values was examined using bi-trait mixed-effect models. Ratio-based behavioral traits, such as the ratio of daily total feeding duration to ruminating duration, were also analyzed.

**Results:**

Estimated heritability were generally low, ranging from 0.02 to 0.37, accounting for 22.7%–67.5% of the repeatability. Genetic correlations between behavioral and carcass traits were also low in magnitude (− 0.28 to 0.29); nevertheless, significant correlations were detected for all carcass traits except rib thickness. These correlations were interpretable; for example, animals that fed and moved less frequently and for shorter durations, and that ruminated more frequently and for longer durations, exhibited greater carcass weight. Ratio-based behavioral traits were heritable and often showed significant genetic correlations with carcass traits, which aided in the interpretation of the results. Incorporating behavioral traits as secondary traits into bi-trait mixed-effect models did not improve the accuracy of carcass breeding value predictions, likely due to the low heritability and weak genetic correlations observed.

**Conclusions:**

Although heritability and genetic correlations were generally low, the findings provide novel insights into the genetic relationships between behavioral and carcass traits. These results offer fundamental information that may support the use of behavioral traits in breeding and management strategies for beef cattle.

**Supplementary Information:**

The online version contains supplementary material available at 10.1186/s12711-026-01082-5.

## Background

Feeding and rumination behavior of cattle have been investigated for breeding purposes because these behavioral traits are associated with feed intake and efficiency. For example, in Nellore cattle, the duration of a single feeding event shows positive genetic correlations with residual feed intake (RFI) and average daily gain [1]. Feeding behavior has also been reported to be genetically and phenotypically correlated with feed efficiency and growth traits in other beef cattle populations [2–9] and dairy cattle populations [10]. Rumination time is genetically correlated with RFI and dry matter intake (DMI) [11, 12] and phenotypically associated with DMI [13] in Holstein cows. Therefore, feeding and rumination behavior can serve as selection targets to improve feed efficiency.

In contrast, less attention has been paid to other behavioral traits such as lying, standing, and moving. Thus, the genetic parameters of these traits and their relations to economic traits remain largely unclear. Moreover, genetic correlations between behavioral traits and carcass traits have been less characterized than those involving feed efficiency and growth traits. Although several studies have reported genetic correlations between feeding behavior and carcass traits [14–16], more evidence is required to understand the genetic relationships between behavioral and carcass traits in beef cattle, particularly considering that genetic influence may differ among breeds [14].

Genomic evaluation using genome-wide DNA markers is now standard in livestock industries [17–19]. Its main advantage over pedigree-based evaluation is the superior prediction accuracy of breeding values. Multi-trait models that incorporate traits genetically correlated with focal traits as secondary traits can further enhance prediction accuracy [20–22]. Because behavioral traits can be measured during the juvenile stages of candidate bulls, they have the potential to serve as informative secondary traits for predicting the breeding values of carcass traits in beef cattle.

To gain insight into beef cattle behavior and identify informative secondary traits, we also analyzed ratios of behavioral traits (e.g., relative duration of feeding against ruminating or relative frequency of feeding vs. moving). The ratios between rumination and activity or feeding time have been associated with feed conversion efficiency in dairy cattle [23]. The ratios between rumination and feeding have been reported to change before mastitis [24]. These results suggest that ratios between behaviors are related to animal physiology and economic traits; however, comprehensive surveys have not been conducted for beef cattle.

In this study, we analyzed seven behavioral classes—feeding, lying, moving, standing, ruminating, ruminating while lying, and ruminating while standing—measured using wearable sensors in Japanese Black cattle. From consecutive daily observations, we calculated six behavioral metrics for each behavioral class: daily total duration, daily frequency, average duration, longest duration, average interval, and longest interval. In addition, ratios between behavioral classes were calculated for each metric and included in the analysis. The objectives of this study are (1) to estimate the heritability and repeatability of these behavioral traits, (2) to estimate the genetic correlations between the behavioral and carcass traits, and (3) to evaluate the multi-trait mixed-effect models that include behavioral traits as secondary traits for predicting the breeding values of carcass traits.

## Methods

### Animals

Japanese black cattle behavior was automatically measured using U-motion (DESAMIS Co., Ltd., Tokyo, Japan), a real-time monitoring system that classifies cattle activity into six behavioral classes—feeding, lying, moving, standing, ruminating while lying, and ruminating while standing—based on acceleration and barometric sensor outputs accumulated within a 10 min window. Although the reliability of U-motion has not yet been evaluated in scientific literature, a third-party assessment conducted by SEGES Innovation, an agricultural research institute in the Kingdom of Denmark, tested the system on 655 cows to verify its effectiveness for heat and disease detection. The institute reported that U-motion can detect heat in cows and identify pregnant cows [25]. In addition, by July 2024, over 200,000 units of U-motion had been sold in Japan, demonstrating its widespread adoption [26]. Thus, we consider U-motion as a reliable device that offers the measurement accuracy required for scientific research.

Behavioral monitoring was conducted at two research stations of the Livestock Improvement Association of Japan (LIAJ), located in Hiroshima (N34.428 and E132.886) and Hokkaido (N42.895 and E143.333) prefectures. LIAJ conducted progeny testing using these stations. All the animals included in this study were progenies of young candidate sires. Progeny testing of LIAJ utilizes a staggered cohort design, in which progeny from different candidate sires are introduced into the research stations at 6-month intervals. The animals were raised for approximately 30 (heifers) or 28 (steers) months and slaughtered. Behavioral monitoring was conducted between June 2021 and March 2024 (1037 days), and 1032 animals were monitored for an average of 439 ± 186 days. Of the 1032 animals, 544 finished the program during the monitoring period and were slaughtered. A summary of the behavioral and carcass data is presented in Tables 1 and 2, respectively. All management conditions reported in our previous study [27] are also presented in Table 1.

**Table 1:** Summary of behavioral data from each research station

Location	Hiroshima	Hokkaido
	N34.428 and E132.886	N42.895 and E143.33
Mean daily temperature (℃)^a^	15.3 ± 8.3	9.3 ± 9.9
Mean daily relative humidity (%)^b^	80.3 ± 7.7	76.1 ± 8.1
Number of heifers	215	199
Number of steers	367	251
Age of starting measurement (days)	268.3 ± 31.2	291.8 ± 87.6
Measurement period per animal (days)	431.5 ± 204.1	476.9 ± 171.6
Total number of measurements	36,122	30,376
Number of sires	101	86
Number of common sires between stations	73	
Number of animals per sire	5.8 ± 3.1	5.2 ± 2.3
Herd management	5–6 animals/pen following a 2-week prevention period with 10 animals/pen. All pens were inside barns.	15 animals/pen until 14 months and 3 to 4 animals/pen thereafter. All pens were inside barns.
Access to diet and water	Ad libitum	Ad libitum
Feeding time	0830–0900 and 1530–1600	0900 and 1600
Forage: concentrate ratio	56:44 at the beginning of fatting and 13:87 during fatting	56:44 at the beginning of fatting and 13:87 during fatting
Target average daily gain (kg/day)	0.85	0.80
Forage	Timothy until 12 months and barley stalk after 11 months	Timothy until 13 months and rice stalk thereafter
Concentrate diet	Grains, brans, oilcake, and others with > 73% total digestible nutrients and > 12% crude protein	Grains, brans, oilcake, and others with > 73% total digestible nutrients and > 12% crude protein

^a^Averages of the mean daily temperature from June 2021 to March 2024

^b^Averages of the mean daily relative humidity from June 2021 to March 2024

**Table 2:** Summary of carcass records from each research station^a^

Number of heifers with carcass records	Hiroshima	Hokkaido
	110	112
Number of steers with carcass records	175	147
Age of slaughter of heifers (days)	907.9 ± 46.2	905.1 ± 29.3
Age of slaughter of heifers (days)	841.3 ± 42.4	845.8 ± 22.5
Number of sires of animals with carcass records	42	46
Number of common sires between stations	33	
Number of animals per sire	6.8 ± 3.2	5.6 ± 2.3

^a^These animals are a part of those listed in Table 1

### Behavior trait definitions

**Fig. 1:**
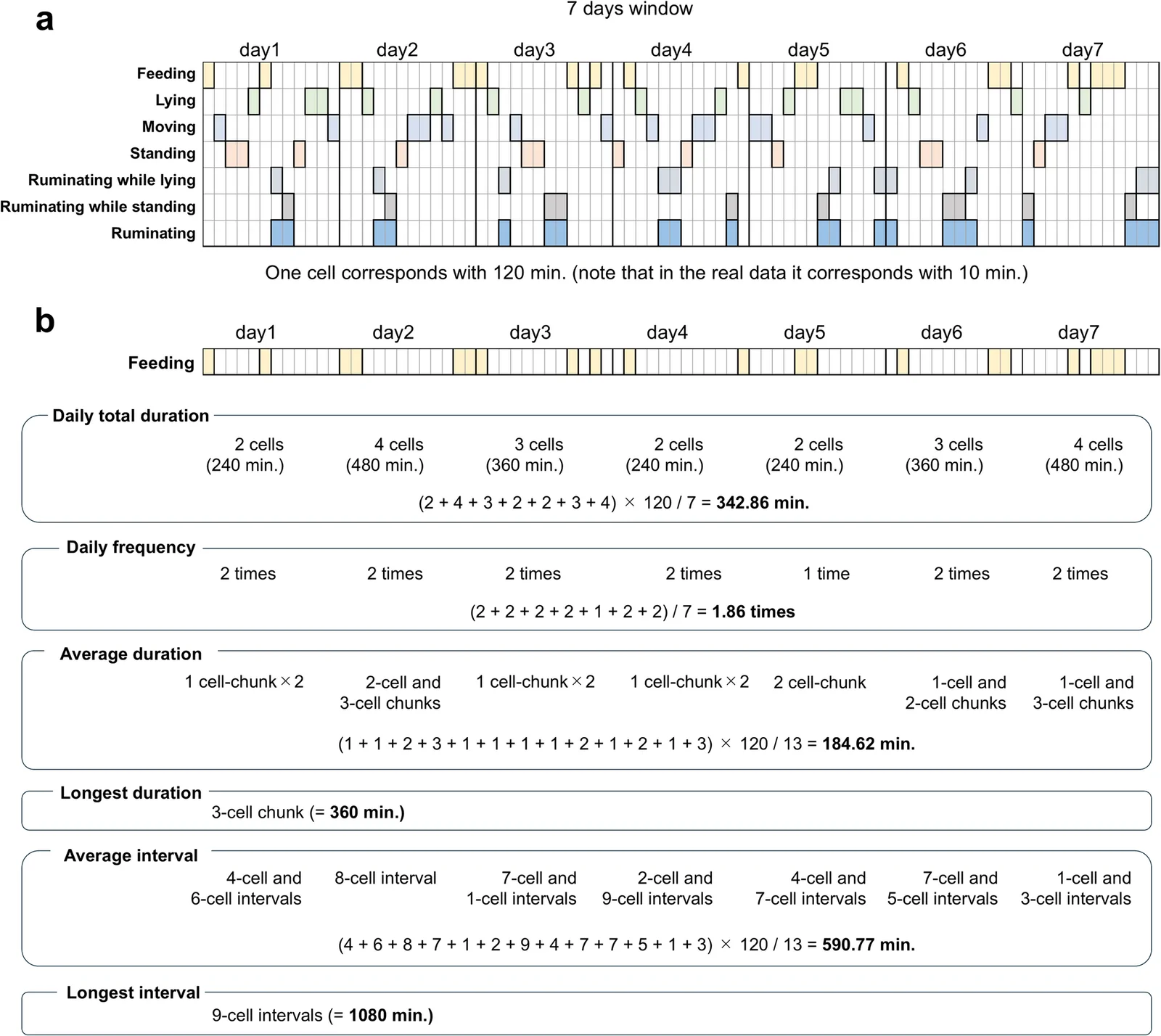
Illustration of trait definitions. **a** An illustration of monitoring results for an animal. Records in a 1-week window are shown. U-motion classifies cattle behavior into six classes (feeding, lying, moving, standing, ruminating while lying, and ruminating while standing) every 10 min using sensor outputs. Ruminating is defined as the combination of ruminating while lying and standing. Each animal has 144 (6 $\:\times\:$ 24) records per day. In this figure, for illustration purposes, the animal has 12 records per day, supposing that behavior is classified every 120 min. Note that this illustration is just an example and may be unrealistic (e.g., feeding behavior usually concentrated around feeding times). **b** An illustration of the definitions of six behavioral metrics (daily total duration, daily frequency, average duration, longest duration, average interval, and longest interval). Feeding behavior in (a) is used as an example.

For each animal, behavior was recorded every 10 min as one of the six predefined classes, resulting in each animal having 144 (6 $\:\times\:$ 24 h) records per day. Using these records, we defined six behavioral trait metrics—daily total duration, daily frequency, average duration, longest duration, average interval, and longest interval—for each behavioral class. The definitions are illustrated in Fig. 1a and b. First, the entire monitoring period (June 2021–March 2024) was divided into 148 nonoverlapping windows. Each window covered 1 week, except for the last window, which was 8 days long. As illustrated in Fig. 1a, each window contained multiple chunks, defined as sequences of the same behavior. For each window, the phenotypic values of the six behavioral metrics were calculated as follows.

- Daily total duration: average total duration of the behavioral class per day.
- Daily frequency: average number of behavior chunks per day.
- Average duration: mean length of behavior chunks in the window.
- Longest duration: maximum behavior chunk length in the window.
- Average interval: mean time interval between consecutive behavior chunks in the window.
- Longest interval: maximum interval between consecutive chunks in the window.

In addition to the six behavior classes (feeding, lying, moving, standing, ruminating while lying, and ruminating while standing), we created an additional class, “ruminating,” by combining ruminating while lying and ruminating while standing. As a result, we obtained seven behavioral classes and 42 traits (7 classes $\:\times\:$ 6 metrics). Furthermore, we calculated ratio-based traits by taking the ratios between two classes, e.g., the ratio of daily total duration for feeding to that for moving. The number of combinations of behavior classes was 21 (7 $\:\times\:$ 6 ÷ 2). Thus, the number of ratio-based traits was 126 (21 $\:\times\:$ 6 metrics). Consequently, the total number of behavioral traits analyzed was 168 (42 + 126). When calculating ratios between classes, if the phenotypic value of the denominator class is 0, the ratio is infinite, whereas the ratio is finite (0) when the numerator class is 0. To avoid this asymmetry, ratios were treated as NA (missing value) if either the denominator or numerator was 0. Preliminary analyses revealed that the estimated genetic parameters for the ratio-based traits were minimally affected by the choice of class used as the denominator. Thus, the denominator traits were selected arbitrarily. Descriptive statistics for the main behavioral traits (i.e., 42 non-ratio traits) are listed in Table 3. The 126 ratio traits are listed in [Additional file 1, Table S1]. The distributions of phenotypic values are shown in [Additional file 2, Figs. S1 and S2].

**Table 3:** Descriptive statistics and narrow-sense heritability of behavioral traits^a^

Trait metric	Behavioral class	Mean (SD)	Range	Heritability (SE)^b^
Daily total duration (min/day)	Feeding	350.4 (124.1)	1.4–1035.0	0.37 (0.03)
	Moving	159.6 (81.5)	1.4–792.9	0.18 (0.03)
	Lying	398.5 (98.7)	45.0–1357.1	0.22 (0.03)
	Standing	192.0 (64.3)	7.1–1125.7	0.21 (0.03)
	Ruminating while lying	256.5 (77.4)	1.4–637.4	0.14 (0.03)
	Ruminating while standing	83.2 (39.6)	1.4–437.1	0.24 (0.03)
	Ruminating	339.7 (85.5)	7.1–721.4	0.16 (0.03)
Daily frequency	Feeding	10.2 (2.1)	0.1–25.9	0.14 (0.02)
	Moving	11.7 (4.5)	0.1–33.0	0.14 (0.03)
	Lying	14.4 (2.5)	0.9–30.3	0.12 (0.02)
	Standing	12.2 (3.3)	0.0–30.2	0.23 (0.03)
	Ruminating while lying	11.8 (2.5)	0.0–25.1	0.11 (0.02)
	Ruminating while standing	5.9 (2.4)	0.0–24	0.27 (0.03)
	Ruminating	16.1 (2.9)	0.0–36.9	0.19 (0.03)
Average duration (min per week)	Feeding	35.6 (15.6)	10.0–231.8	0.33 (0.03)
	Moving	13.2 (2.0)	10.0–35.7	0.17 (0.02)
	Lying	28.4 (16.7)	10.0–1336.7	0.16 (0.03)
	Standing	15.9 (13.4)	0.0–2433.3	0.07 (0.02)
	Ruminating while lying	21.5 (4.7)	0.0–60.0	0.19 (0.03)
	Ruminating while standing	14.0 (2.1)	0.0–60.0	0.07 (0.01)
	Ruminating	21.1 (4.5)	0.0–50.1	0.20 (0.03)
Longest duration (min per week)	Feeding	156.4 (82.5)	10.0–880.0	0.27 (0.03)
	Moving	42.2 (19.4)	10.0–310.0	0.11 (0.02)
	Lying	112.1 (135.8)	10.0–9230.0	0.13 (0.02)
	Standing	70.9 (110.2)	0.0–5630.0	0.06 (0.01)
	Ruminating while lying	59.2 (15.2)	0.0–240.0	0.14 (0.02)
	Ruminating while standing	40.1 (13.7)	0.0–390.0	0.07 (0.01)
	Ruminating	63.8 (16.3)	0.0–390.0	0.15 (0.02)
Average interval (min per week)	Feeding	113 (69.9)	22.0–5035.0	0.21 (0.03)
	Moving	129.4 (72.5)	22.8–5035.0	0.15 (0.03)
	Lying	75 (19.7)	19.0–1122.9	0.16 (0.03)
	Standing	111.1 (42.7)	32.9–1908.0	0.23 (0.03)
	Ruminating while lying	107.5 (77.2)	34.0–10080.0	0.12 (0.03)
	Ruminating while standing	278.1 (205.1)	46.2–10080.0	0.27 (0.03)
	Ruminating	72.2 (57.7)	22.0–10080.0	0.18 (0.03)
Longest interval (min per week)	Feeding	724.7 (396.1)	40.0–9460.0	0.16 (0.02)
	Moving	723.3 (427.4)	70.0–9420.0	0.09 (0.02)
	Lying	505.9 (210.7)	60.0–5650.0	0.04 (0.01)
	Standing	577.1 (246.6)	120.0–9230.0	0.12 (0.02)
	Ruminating while lying	651.8 (433.1)	100.0–10080.0	0.04 (0.01)
	Ruminating while standing	1183.5 (657.8)	150.0–10080.0	0.18 (0.02)
	Ruminating	424.3 (397.4)	60.0–10080.0	0.07 (0.01)

^a^For each trait, the number of records (weakly averages) was 66,498 except for standing (66,493), ruminating while lying (66,494), ruminating while standing (66,455), and ruminating (66,452) of daily total duration. The number of animals was 1032 for each trait

^b^Variance components are listed in [Additional file 1, Table S2]

### Carcass traits

Among the 1032 animals with behavioral traits, 544 were slaughtered and evaluated for six carcass traits: carcass weight (CW, kg), rib eye area (REA, cm^2^), rib thickness (RT, cm), subcutaneous fat thickness (SFT, cm), yield rate (YI, %), and beef marbling score (BMS). Carcass traits were assessed by the Japan Meat Grading Association, the official grader in Japan. BMS ranged from 1 to 12, with higher values indicating more abundant intramuscular fat. Descriptive statistics for carcass traits are presented in Table 4.

**Table 4:** Descriptive statistics and narrow-sense heritability of carcass traits^a^

Trait	Unit	Mean (SD)	Range	Heritability (SE)^b^
Carcass weight	kg	492.2 (57.1)	259.8–650.0	0.72 (0.08)
Rib eye area	cm^2^	65.3 (11.2)	35.0–109.0	0.47 (0.09)
Rib thickness	cm	7.9 (0.9)	3.6–10.6	0.44 (0.09)
Subcutaneous fat thickness	cm	2.5 (0.7)	1.0–5.0	0.49 (0.09)
Yield rate	%	74.8 (1.6)	70.5–80.8	0.46 (0.09)
Beef marbling score	Score between 1 and 12	8.1 (2.3)	3–12	0.45 (0.09)

^a^The number of animals was 544 for each trait

^b^Variance components are listed in [Additional file 1, Table S4]

### SNP genotyping

The 1032 animals were genotyped using an Illumina SNP Chip customized by LIAJ (ver. 1.0). After pruning SNPs with call rates < 0.9 or minor allele frequencies < 0.05, 36,572 from the 50,019 SNPs autosomal SNPs were selected for analysis. Missing genotypes were imputed using Beagle ver. 5.3 [28]. Subsequently, a genomic relationship matrix was created using the R package rrBLUP [29] based on the first method of VanRaden [30] and used to estimate variance components.

### Transformation

The phenotypic values of the 168 behavioral traits often did not follow a normal distribution [Additional file 2, Figs. S1 and S2]. Our previous study showed that Poisson regression models were preferable to repeatability models with normal assumptions for U-motion–derived behavioral traits [27]. However, applying Poisson regression to 168 traits would be computationally intensive. Thus, a Box–Cox transformation was applied to each behavior trait using the R package “car” (ver. 3.1-3) [31]. The distributions of transformed phenotypic values are shown in [Additional file 2, Figs. S3 and S4]. After transformation, we removed all values that were outside the range defined by the following criterion: mean ± 3 standard deviations (SDs).

### Single-trait analysis

In the single-trait analysis, repeatability models were applied to the transformed phenotypic values of each behavior trait. The models can be written as follows:

1 $$ y_{{i,w}} = {\mathbf{x}}_{{{\mathrm{i}},{\mathrm{w}}}} ^{{\mathrm{T}}} {\mathbf{\beta }} + u_{i} + pe_{i} + e_{{i,w}} , $$

where $\:{y}_{i,w}$ is the transformed phenotypic value of animal * i* in window * w*, $\:{\mathbf{x}}_{\mathrm{i},\mathrm{w}}$ is the column vector of fixed effects, T indicates transpose, $\:\boldsymbol{\upbeta\:}$ is the column vector of regression coefficients, $\:{u}_{i}$ is the additive genetic effect of animal * i*, $\:{pe}_{i}$ is the permanent environmental effect of animal * i*, and $\:{e}_{i,w}$ is the residual. $\:{\mathbf{x}}_{\mathrm{i},\mathrm{w}}$ included the intercept, sex (steer or heifer), month of monitoring (12 months), year of monitoring (4 years), farm (Hokkaido or Hiroshima), average age in window *w* (days) and its quadratic term, and the average temperature humidity index (THI) for window *w* and its quadratic form. Temperature and humidity in the barns were recorded using indoor sensors, and THI was calculated according to a previous study [32]. The covariance structure of the additive genetic effect was defined using the genomic relationship matrix, and residuals were assumed to follow an identity covariance structure.

For the carcass traits, animal models were applied separately to each trait:

2 $$\:{y}_{i}={{\mathbf{x}}_{\mathrm{i}}}^{\mathrm{T}}\boldsymbol{\upbeta\:}+{u}_{i}+{e}_{i},$$

where $\:{y}_{i}$ is the phenotypic value of animal * i*, $\:{\mathbf{x}}_{\mathrm{i}}$ is the column vector for fixed effects, $\:\boldsymbol{\upbeta\:}$ is the column vector of regression coefficients, $\:{u}_{i}$ is the additive genetic effect of animal * i*, and $\:{e}_{i}$ is the residual. $\:{\mathbf{x}}_{\mathrm{i}}$ included the intercept, sex (steer or heifer), month of slaughter (12 months), year of slaughter (2022, 2023, or 2024), farm (Hokkaido or Hiroshima), and slaughter age (days) and its quadratic term. The covariance structure of the additive genetic effect was defined using the genomic relationship matrix. The covariance structure of the residuals was the identity matrix.

The single-trait analyses were performed using airemlf90 (ver. 1.149) [33]. Each trait was analyzed 10 times using different initial values. When the solutions across the 10 replicates were inconsistent, solutions supported by most of the replicates were adopted. We used the default value of the convergence criteria.

### Bi-trait analysis 1

To estimate the genetic correlation between behavioral non-ratio traits, bi-trait models were applied to each combination of non-ratio traits. Ratio traits were excluded from the analyses to reduce the computational burden. Combinations between 42 traits (7 classes $\:\times\:$ 6 metrics) were considered. The bi-trait model for two behavioral traits, * b1* and * b2*, was expressed as follows:

3 $$\begin{aligned} \left[ {\begin{array}{*{20}c} {y_{{b1,i,w}} } \\ {y_{{b2,i,w}} } \\ \end{array} } \right] = & \left[ {\begin{array}{*{20}c} {{\mathbf{x}}_{{b1,i,w}}^{T} } & {0} \\ 0 & {{\mathbf{x}}_{{b2,i,w}}^{T} } \\ \end{array} } \right]\left[ {\begin{array}{*{20}c} {{\mathbf{\beta }}_{{b1}} } \\ {{\mathbf{\beta }}_{{b2}} } \\ \end{array} } \right] \\ & + \left[ {\begin{array}{*{20}c} {u_{{b1,i}} } \\ {u_{{b2,i}} } \\ \end{array} } \right] + \left[ {\begin{array}{*{20}c} {pe_{{b1,i}} } \\ {pe_{{b2,i}} } \\ \end{array} } \right] + \left[ {\begin{array}{*{20}c} {e_{{b1,i,w}} } \\ {e_{{b2,i,w}} } \\ \end{array} } \right], \\ \end{aligned}$$

where $\:{y}_{b1,i,w}$ and $\:{y}_{b2,i,w}$ are the phenotypic values of behavioral traits * b1* and * b2* for animal * i* in window * w*. $\:{\mathbf{x}}_{\mathrm{b}1,i,w}$ and $\:{\mathbf{x}}_{b2,i,w}$ are the column vectors for the fixed effects, which are the same as those used in Eq. (1). $\:{\boldsymbol{\upbeta\:}}_{b1}$ and $\:{\boldsymbol{\upbeta\:}}_{b2}$ are the column vectors for the regression coefficients, $\:{u}_{b1,i}$ and $\:{u}_{b2,i}$ are the additive genetic effects of animal * i*, $\:{pe}_{b1,i}$ and $\:{pe}_{b2,i}$ are the permanent environmental effects, and $\:{e}_{b1,i,w}$ and $\:{e}_{b2,i,w}\:$are the residuals. The additive genetic effects, permanent environmental effects, and residuals followed multivariate normal distributions as follows:

4 $$\:\left[\begin{array}{c}{u}_{b1,i}\\\:{u}_{b2,i}\end{array}\right]\sim\mathrm{M}\mathrm{V}\mathrm{N}\left(\begin{array}{cc}0&\:{\boldsymbol{\Sigma\:}}_{u,b1,b2}^{2}\end{array}\right),$$

5 $$\:\left[\begin{array}{c}{pe}_{b1,i}\\\:{pe}_{b2,i}\end{array}\right]\sim\mathrm{M}\mathrm{V}\mathrm{N}\left(\begin{array}{cc}0&\:{\boldsymbol{\Sigma\:}}_{pe,b1,b2}^{2}\end{array}\right),$$

and

6 $$\:\left[\begin{array}{c}{e}_{b1,i,w}\\\:{e}_{b2,i,w}\end{array}\right]\sim\mathrm{M}\mathrm{V}\mathrm{N}\left(\begin{array}{cc}0&\:{\boldsymbol{\Sigma\:}}_{e,b1,b2}^{2}\end{array}\right),$$

respectively, where $\:{\boldsymbol{\Sigma\:}}_{u,b1,b2}^{2}$, $\:{\boldsymbol{\Sigma\:}}_{pe,b1,b2}^{2}$, and $\:{\boldsymbol{\Sigma\:}}_{e,b1,b2}^{2}$ are the additive genetic, permanent environment, and residual variance–covariance matrices between the behavioral traits * b1* and * b2*. The additive genetic covariance structure among animals was defined using the genomic relationship matrix. These bi-trait analyses were conducted using airemlf90 (ver. 1.149). Each trait combination was analyzed 10 times using different initial values. When the solutions among the 10 replicates were inconsistent, the solutions supported by most of the replicates were adopted. The default value of the convergence criteria was used.

### Bi-trait analysis 2

To estimate the genetic and residual correlations between behavioral and carcass traits, bi-trait models were applied for each combination of behavioral and carcass traits. Because the behavioral and carcass phenotypes did not correspond to one another (i.e., each animal has one phenotypic value for carcass traits, while each animal has multiple phenotypic values for behavioral traits), bi-trait models could not be applied. Thus, the transformed phenotypic values of each behavioral trait were first adjusted for the fixed and permanent environment effects estimated in the single-trait models. These adjusted phenotypic values were then averaged for each animal, yielding one phenotypic value per trait per animal. Using these phenotypic values, the bi-trait model was written as follows:

7 $$ \begin{aligned} \left[ {\begin{array}{*{20}c} {\tilde{y}_{{b,i}} } \\ {y_{{c,i}} } \\ \end{array} } \right] = & \left[ {\begin{array}{*{20}c} {{\mathbf{x}}_{{{\mathrm{b}},{\mathrm{i}}}}^{T} } & 0 \\ 0 & {{\mathbf{x}}_{{{\mathrm{c}},{\mathrm{i}}}}^{T} } \\ \end{array} } \right]\left[ {\begin{array}{*{20}c} {{\mathbf{\beta }}_{{\mathrm{b}}} } \\ {{\mathbf{\beta }}_{{\mathrm{c}}} } \\ \end{array} } \right] \\ & \quad + \left[ {\begin{array}{*{20}c} {u_{{b,i}} } \\ {u_{{c,i}} } \\ \end{array} } \right] + \left[ {\begin{array}{*{20}c} {e_{{b,i}} } \\ {e_{{c,i}} } \\ \end{array} } \right], \\ \end{aligned} $$

where $\:{\stackrel{\sim}{y}}_{b,i}$ is the adjusted and averaged phenotypic value of behavioral trait *b* of animal * i* and $\:{y}_{c,i}$ is the phenotypic value of carcass trait *c* for animal *i*. $\:{\mathbf{x}}_{\mathrm{b},i}$ and $\:{\mathbf{x}}_{\mathrm{c},i}$ are the column vectors for fixed effects and $\:{\boldsymbol{\upbeta\:}}_{\mathrm{b}}$ and $\:{\boldsymbol{\upbeta\:}}_{\mathrm{c}}$ are the column vectors of regression coefficients. $\:{\mathbf{x}}_{\mathrm{b},i}$ included only the intercept, and $\:{\mathbf{x}}_{\mathrm{c},i}$ was the same as $\:{\mathbf{x}}_{\mathrm{i}}$ in Eq. (2). $\:{u}_{b,i}$ and $\:{u}_{c,i}$ are the additive genetic effects of animal * i*, and $\:{e}_{b,\:i}$ and $\:{e}_{c,\:i}\:$are the residuals. Note that $\:{y}_{c,i}$ included missing values because only 544 of the 1032 animals were phenotyped for carcass traits. The additive genetic effects and residuals were assumed to follow multivariate normal distributions as follows:

8 $$ \begin{aligned} \left[ {\begin{array}{*{20}c} {u_{{b,i}} } \\ {u_{{c,i}} } \\ \end{array} } \right]\sim & {\mathrm{MVN}}\left( {\begin{array}{*{20}c} {0,} & {{\mathbf{\Sigma }}_{u}^{2} } \\ \end{array} } \right)\;{\mathrm{and}} \\ & \left[ {\begin{array}{*{20}c} {e_{{b,i}} } \\ {e_{{c,i}} } \\ \end{array} } \right]\sim {\mathrm{MVN}}\left( {\begin{array}{*{20}c} {0,} & {{\mathbf{\Sigma }}_{e}^{2} } \\ \end{array} } \right), \\ \end{aligned} $$

respectively, where $\:{\boldsymbol{\Sigma\:}}_{u}^{2}$ and $\:{\boldsymbol{\Sigma\:}}_{e}^{2}$ are the additive genetic and residual variance-covariance matrices. The additive genetic covariance structure among animals was defined using the genomic relationship matrix. The covariance structure of the residual $\:{e}_{b,i}$ among animals was written as follows:

9 $$ \left[ {\begin{array}{*{20}c} {e_{{b,1}} } \\ \vdots \\ {e_{{b,N}} } \\ \end{array} } \right]\sim {\mathrm{MVN}}\left( {\begin{array}{*{20}c} {0,} & {{\mathbf{W}}\sigma _{b}^{2} } \\ \end{array} } \right), $$

where $\:\mathbf{W}$ is a diagonal matrix with elements equal to the inverse of the number of records contributing to each animal’s averaged phenotype, and $\:{\sigma\:}_{b}^{2}$ is the first diagonal element of $\:{\boldsymbol{\Sigma\:}}_{e}^{2}$. The covariance structure of the residual $\:{e}_{c,i}$ was the identity matrix.

These bi-trait analyses were conducted in a Bayesian framework using Stan implemented in cmdstanr (ver. 0.8.0) [34]. Stan was adopted because airemlf90 did not accept different weights for each trait included in multi-trait models. For the Stan implementation, the additive genetic and residual variance-covariance matrices $\:{\boldsymbol{\Sigma\:}}_{u}^{2}$ and $\:{\boldsymbol{\Sigma\:}}_{e}^{2}$ were decomposed as follows:

10 $$ \begin{aligned} {\mathbf{\Sigma }}_{u}^{2} = & \left[ {\begin{array}{*{20}c} {\sigma _{{u,b}} } & 0 \\ 0 & {\sigma _{{u,c}} } \\ \end{array} } \right]^{T} \left[ {\begin{array}{*{20}c} 1 & {r_{{u,bc}} } \\ {r_{{u,bc}} } & 1 \\ \end{array} } \right] \\ & \quad \left[ {\begin{array}{*{20}c} {\sigma _{{u,b}} } & 0 \\ 0 & {\sigma _{{u,c}} } \\ \end{array} } \right] \\ \end{aligned},$$

and

11 $$ \begin{aligned} {\mathbf{\Sigma }}_{e}^{2} = & \left[ {\begin{array}{*{20}c} {\sigma _{{e,b}} } & 0 \\ 0 & {\sigma _{{e,c}} } \\ \end{array} } \right]^{T} \left[ {\begin{array}{*{20}c} 1 & {r_{{e,bc}} } \\ {r_{{e,bc}} } & 1 \\ \end{array} } \right] \\ & \quad \left[ {\begin{array}{*{20}c} {\sigma _{{e,b}} } & 0 \\ 0 & {\sigma _{{e,c}} } \\ \end{array} } \right], \\ \end{aligned} $$

where $$\:{\sigma\:}_{u,b}$$ and $$\:{\sigma\:}_{u,c}$$ are the additive genetic standard deviations for the behavioral and carcass traits, $$\:{r}_{u,bc}$$ is the genetic correlation between the behavioral and carcass traits, $$\:{\sigma\:}_{e,b}$$ and $$\:{\sigma\:}_{e,c}$$ are the residual standard deviations, and $$\:{r}_{e,bc}$$ is the residual correlation. The prior distributions of the standard deviations ($$\:{\sigma\:}_{u,b},\:$$$$\:{\sigma\:}_{u,c},\:$$$$\:{\sigma\:}_{e,b},$$ and $$\:{\sigma\:}_{e,c}$$) were half-Cauchy distributions with location parameter 0.0 and scale parameter 0.5. The prior distributions of the correlation matrices were Lewandowski–Kurowicka–Joe distributions with the parameter η set to 2.0. The prior distributions of the fixed effects ($$\:{\boldsymbol{\upbeta\:}}_{\mathrm{b}}$$ and $$\:{\boldsymbol{\upbeta\:}}_{\mathrm{c}}$$) were the normal distributions with a mean of 0 and a standard deviation of 100. The Stan program is available upon request.

The number of iterations was 5000, and the last 1000 iterations were used for estimating posterior distributions. The number of chains was four. Convergence was assessed using the $\:\widehat{R}$ statistic and considered adequate when $\:\widehat{R}\:$< 1.1 for the genetic covariance between behavioral and carcass traits [35]. If convergence was not achieved, the sampler was extended by an additional 5000 iterations using the final parameter values as the initial values. Then, the last 1000 iterations were used for parameter estimation. This process was repeated until convergence. Posterior means of the last 1000 iterations were used as the final estimates.

### ANOVA

Two-way analysis of variance was used to identify the factors that dominate the variance in heritability and reliability estimates. The response variables were the heritability and reliability estimates, and the explanatory factors were the trait classes (feeding, lying, moving, standing, ruminating while lying, ruminating while standing, and ruminating) and the behavioral metrics (daily total duration, daily frequency, average duration longest duration, average interval, and longest interval). Analysis of variance (ANOVA) was conducted using the aov function in R (ver. 4.2.2).

### Cross-validation

To evaluate the accuracy of genomic prediction for carcass traits, 5-fold cross-validation was conducted. Specifically, the 544 animals with carcass trait phenotypes were randomly divided into five groups. In each fold, the phenotypes of one group were masked and predicted from the other groups. This process was repeated for each group. Prediction performance was compared between the single-trait carcass model Eq. (2) and the bi-trait model Eq. (7). When applying the bi-trait model, only the carcass trait phenotypes were masked because the behavioral traits were expected to function as secondary traits. Predicted values were compared with phenotypic values adjusted for fixed effects estimated using the single-trait model Eq. (2) based on 544 animals. Prediction accuracy was measured using the Pearson correlation coefficient between the predicted and adjusted phenotypic values.

Four rules were compared to select behavioral traits as secondary traits.

1. Select the behavioral trait with the highest heritability ($\:{h}^{2}$).
2. Select the behavioral trait showing the highest absolute genetic correlation $\:\left|r\right|$ with the targeted carcass trait.
3. Select the behavioral trait with the highest $\:h\left|r\right|$.
4. Select the behavioral trait that minimized the prediction error variances of the additive genetic effects of the targeted carcass traits, summed across predicted animals.

For rules 1 and 3, the heritability estimated using the single-trait model (Eq. 1) was used. In principle, secondary traits should be selected before prediction. In other words, genetic parameters (variance components and covariances) should be estimated using only training datasets in cross-validation. However, because conducting bi-trait analyses for all combinations of carcass and behavioral traits is quite time-consuming, genetic parameters estimated using all data were used for each fold of cross-validation. Thus, the prediction results may be somewhat optimistic.

## Results and discussion

### Heritability and repeatability

**Fig. 2:**
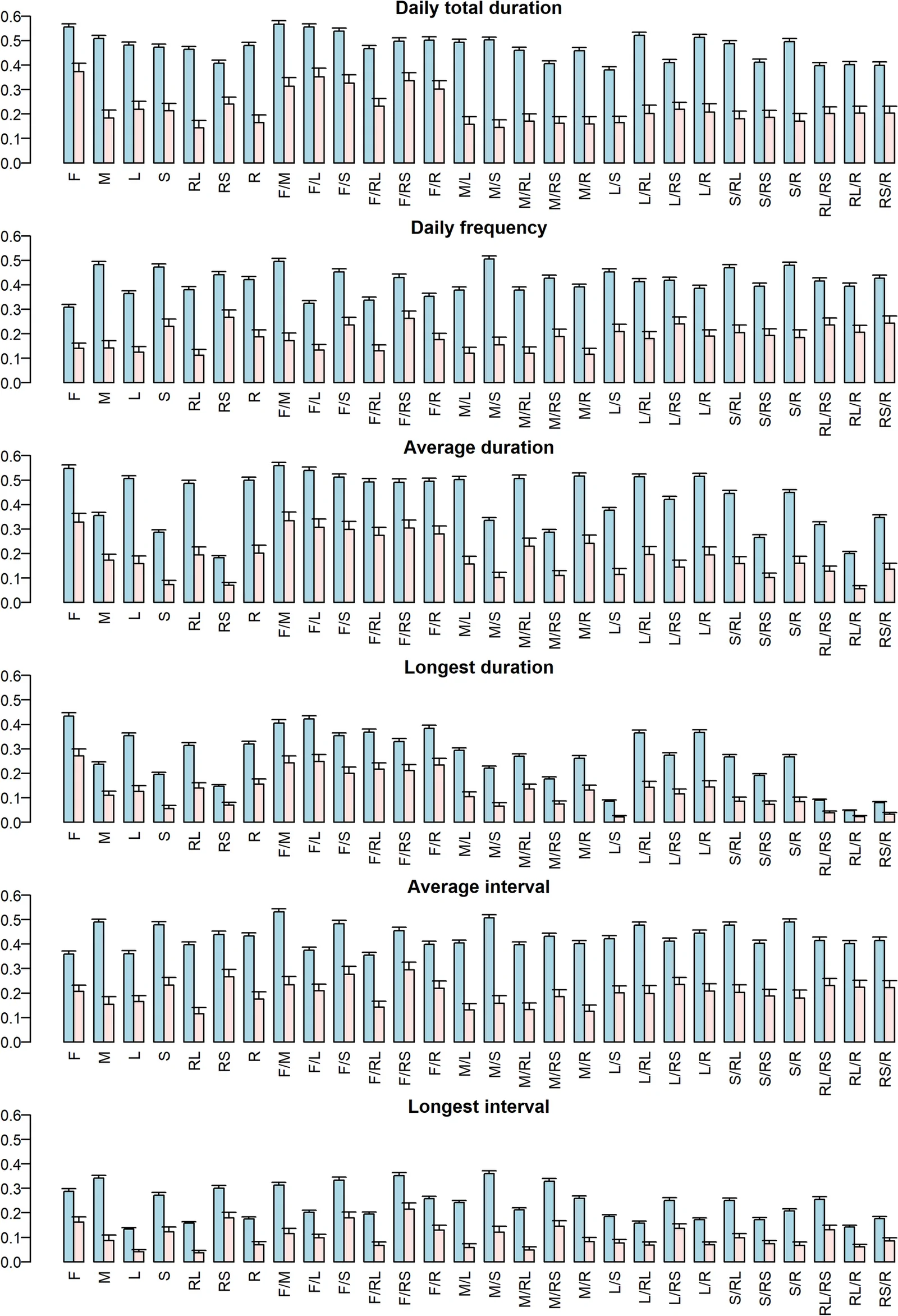
Repeatability and heritability estimates. Blue and pink bars represent repeatability and heritability estimates, respectively. The whiskers indicate the standard errors. Estimates are also presented in [Additional file 1, Tables S2 and S3] along with those of variance components. F: feeding, M: moving, L: lying, S: standing, RL: ruminating while lying, RS: ruminating while standing, R: ruminating. A slash (/) represents the ratios of the numerator traits to the denominator traits

Figure 2 illustrates the repeatability and heritability of behavioral traits. Estimates are also listed in [Additional file 1, Table S2] (behavioral non-ratio traits) and [Additional file 1, Table S3] (behavioral ratio traits) with those of the variance components. Estimates of heritability and variance components for carcass traits are listed in [Additional file 1, Table S4]. Estimates of the heritability of behavioral non-ratio traits and carcass traits are also listed in [Additional file 1, Tables 3 and 4], respectively, with descriptive statistics.

Overall, daily toral duration, daily frequency, average duration, and average interval showed moderate repeatability for each behavior class, with ranges of 0.38–0.57, 0.31–0.51, 0.18–0.56, and 0.35–0.53, respectively. In contrast, the longest duration and longest interval exhibited lower repeatability than those of the four metrics, with ranges of 0.05–0.43 and 0.13–0.36, respectively. Two-way ANOVA revealed that both factors, metric (daily total duration, daily frequency, average duration, longest duration, average interval, and longest interval) and class (feeding, lying, moving, standing, ruminating while lying, ruminating while standing, and ruminating), significantly influenced the variance in repeatability. However, the mean square for metric was substantially larger than that for class (0.261 vs. 0.013) [Additional file, Table S5], indicating that variation in repeatability was more strongly influenced by metric than by class. Heritability accounted for 22.7%–67.5% of the repeatability. Similar to the patterns observed for repeatability, daily toral duration, daily frequency, average duration, and average interval showed higher heritability (ranges: 0.14–0.37, 0.11–0.27, 0.06–0.33, and 0.12–0.30, respectively) than longest duration and longest interval (0.02–0.27 and 0.04–0.21, respectively). Two-way ANOVA also showed that both trait metric and class significantly influenced the variance in heritability, with a larger mean square for metric than for class (0.057 vs. 0.011) [Additional file 1, Table S6].

Comparison of heritability estimates with previous reports is challenging due to differences in various aspects, including sample size, breed composition, management practices, measurement devices, data processing methods, trait definitions, and genetic relationship matrices. Among studies reporting the heritability of cattle behavioral traits [1, 4, 6, 10–12, 14, 16, 36], Cavani et al. [10] are similar to the present study in terms of sample size (1328 in [10] vs. 1032 here) and data processing (weekly averages in both). Major differences include breeds (Holstein cows in [10] vs. Japanese Black steers and heifers here), measurement devices (feeding system vs. accelerometers), and management practices, including pen size (up to 64 animals vs. 3–6 [27]). Cavani et al. [10] calculated meal duration and frequency from feeding events. Their mean meal duration and daily meal frequency were 29.4 min and 7.48 min, respectively, which are close to our measurement statistics for feeding behavior—average duration of feeding (35.6 min) and daily frequency of feeding (10.2) (Table 3). Thus, the traits investigated in [10] correspond to those in the present study. The heritability (repeatability) estimates of these traits in [10] were 0.22 (0.46) and 0.19 (0.51), whereas those in the present study were 0.33 (0.55) and 0.14 (0.31) (Table 3 and Additional file1, Table S2). Although the specific values were slightly different, the overall pattern of low heritability and rich permanent environmental variance was consistent across studies.

Among studies on beef cattle, Kelly et al. [6] reported genetic parameters for feeding behavioral traits measured using automatic feeding stations. Kelly et al. [6] used 1548 animals from multiple breeds (e.g., Limousin and Charolais) and phenotypic values averaged across test days. Similar to the previous study [10], the authors calculated total meal time per day (meal duration, min), number of meals per day (meal frequency), and meal duration (min) from feeding events. These traits correspond to the daily total duration, daily frequency, and average duration of feeding in the present study. The phenotypic values of these traits were largely consistent with our results: the average phenotypic values of these traits in [6] were 213.4, 8.14, and 26.7 min, respectively, whereas those in the present study were 350.4, 10.2, and 35.6 min, respectively (Table 3). Heritability estimates were also consistent between [6] and the present study: 0.49, 0.19, and 0.35, respectively, in [6] vs. 0.37, 0.14. and 0.33, respectively, in our study (Table 3). The slightly higher estimates in [6] may be attributable to rich genetic variation due to multiple breeds and differences in data processing (using daily averages instead of weekly averages).

Another study on beef cattle has reported comparable results. Kava et al. [4] measured the average meal duration and daily meal frequency in Nellore cattle using feeding systems. The phenotypic means were 1.28 min and 7.29 meals/day, respectively. Although the average meal duration was much lower than those in [6, 10], and our study (29.4, 26.7, and 35.6 min respectively), the meal frequency was consistent across studies (7.48, 10.2, and 8.14, respectively). The reported heritability was 0.29 for average meal duration and 0.65 for meal frequency. Repeatability was not reported, probably because the phenotypic values were averaged to yield a single record per animal. The heritability estimate for meal frequency was higher in [4] than in [6, 10] and our study, whereas that for average meal duration was close to those in [6, 10] and our study.

Chen et al. [14] and Benfica et al. [1] also reported heritability estimates for feeding behaviors, duration per feeding event (min/event), frequency of feeding events per day, and total feeding time per day (min/day) in beef cattle, which were measured using feeding systems. These studies used averaged phenotypic values for each animal and reported heritability estimates in the ranges of 0.19–0.40 (duration per feeding event), 0.24–0.43 (feeding frequency per day), and 0.27–0.61 (total feeding time per day). However, comparisons with the present study are difficult because feeding events measured by feeding systems do not necessarily correspond to meals. Overall, the heritability estimates for feeding behavior obtained in the present study were largely consistent with those reported in previous studies [4, 6, 10].

Heritability and repeatability of daily rumination time have been reported in Holstein cows [11, 12, 36]. Using daily records, Byskov et al. [11] estimated the heritability and repeatability of rumination time (min/day) as 0.30–0.33 and 0.75–0.80, respectively. Using weekly averages, Nascimento et al. [12] reported corresponding estimates of 0.25 and ~ 0.81, and Moretti et al. [36] reported the heritability as 0.31–0.36 using daily averages. In comparison, the estimates in our study were 0.16 and 0.48, respectively (Table 3 and Additional file 1, Table S2), which were lower than those in the previous studies. Nascimento et al. [12] also reported heritability and repeatability for lying time (min/day) as 0.48 and 0.84, respectively. The corresponding estimates in our study were 0.22 and 0.48, respectively (Table 3 and Additional file 1, Table S2), which were also lower than those in the previous study. Because Nascimento et al. [12] used accelerometers, Moretti et al. [36] used microphone-based sensors, and Byskov et al. [11] used both depending on herd, the differences in estimates are not expected to be due to measurement methods and may be due to differences in breeds or management practices. For behavior classes other than feeding and ruminating, lying time (min/d) was the only trait with available estimates in the literature [12]. No previous reports were found for the remaining behavioral classes and trait metrics.

In the present study, behavioral records spanning more than 420 days per animal were available for analysis (Table 1). Because the analyses were based on weekly averaged records, this corresponded to approximately 60 repeated observations per animal. In general, a large number of repeated observations is expected to reduce residual variance in a repeatability model and thereby improve the precision of genetic parameter estimates. However, records collected over extended periods inevitably capture temporal changes in animal behavior, which may reduce the suitability of the assumptions underlying a simple repeatability model. Consequently, long-term recording may exert both beneficial and adverse effects on model performance. Future studies should therefore evaluate analytical approaches that more effectively accommodate longitudinal changes in behavioral traits. For example, random regression models incorporating age as a covariate could account for temporal variation in breeding values and provide a more appropriate framework for analyzing long-term behavioral records.

### Genetic correlations between behavioral traits

**Fig. 3:**
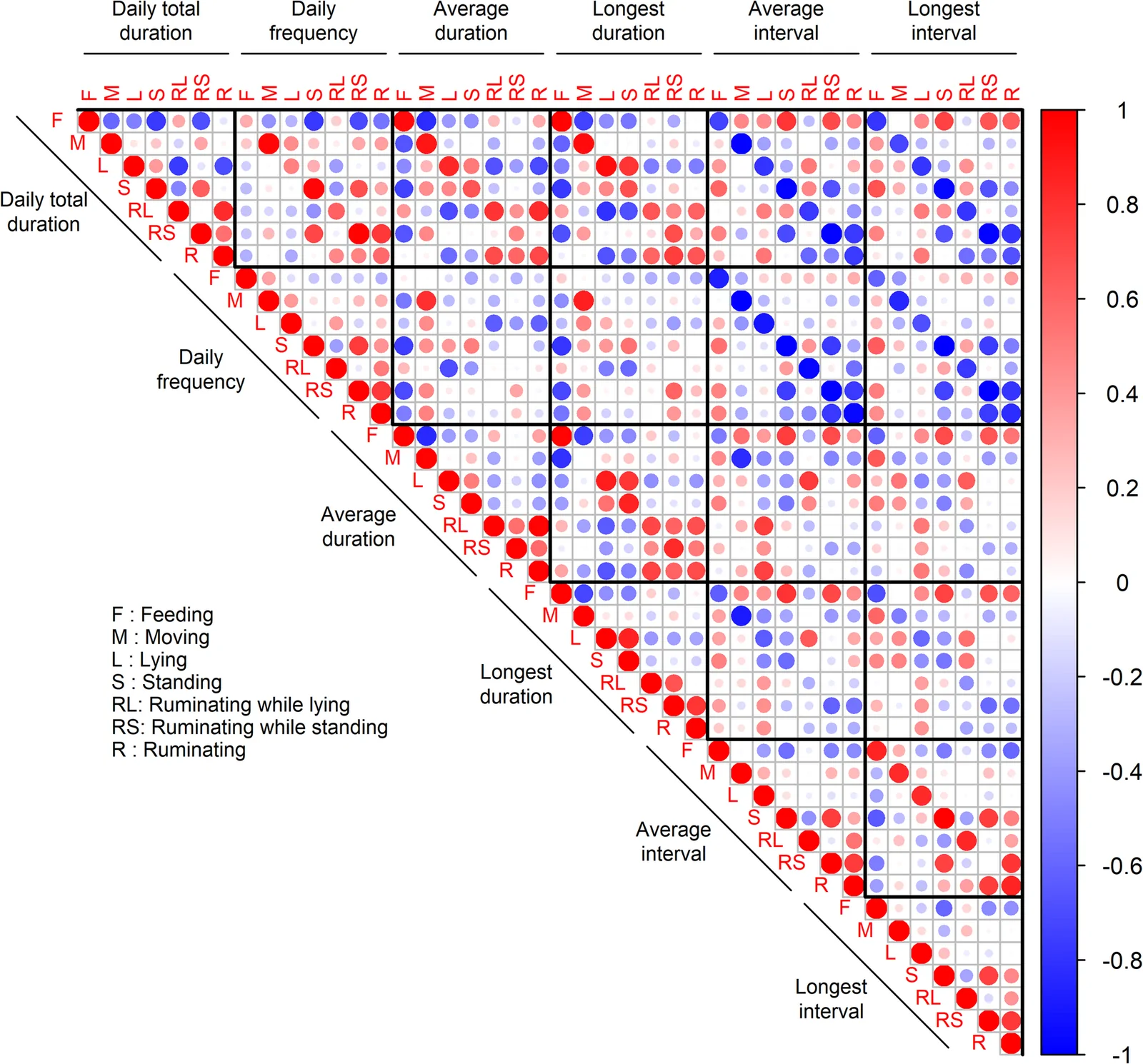
Genetic correlations between behavioral traits. Estimates of the genetic correlation between 42 behavioral non-ratio traits are presented using colors and circles. Positive and negative correlations are presented as red and blue, respectively. The size of the circles indicates the absolute value of the estimate

Estimates of the genetic correlations between behavioral non-ratio traits are shown in Fig. 3. Estimates are also listed in [Additional file 1, Table S7] with phenotypic correlations. As expected from the definitions, traits of daily total duration generally exhibited positive genetic correlations with the traits of the same class of daily frequency and average duration. Similar tendencies were also observed between the average and longest durations and between the average and longest intervals. In contrast, traits of daily total duration exhibited negative correlations with the traits of the same class of average interval and longest interval. Similar tendencies were observed between daily frequency and average and longest interval.

Thus far, genetic correlations between behavioral traits have been examined primarily focusing on feeding behavior. The estimates appear less consistent among studies compared with those of heritability. Cavani et al. [10] estimated the genetic correlation between the number of meals per day and the duration of each meal to be − 0.75; however, Kava et al. [4] estimated the correlation between meal frequency and average meal duration to be 0.03. Our estimate for the daily frequency and average duration of feeding was low (0.067, Fig. 3 and Additional file 1, Table S7). Similarly, Chen et al. [14] estimated the genetic correlation between daily feeding duration and average feeding duration to be 0.29 (Angus) and 0.20 (Charolais), whereas the corresponding estimates of Kava et al. [4] and our study were 0.73 and 0.955, respectively. The estimate from Kava et al. [4] for the correlation between meal frequency and total meal duration was low (− 0.07), whereas our estimate for the daily frequency and daily total duration of feeding was moderate (0.328, Fig. 3 and Additional file 1, Table S7). Inconsistencies among studies are also observed for the genetic correlations between behavioral and carcass traits, as described later.

### Genetic correlations with carcass traits

Significant genetic correlations, defined as those with 95% highest density intervals (HDIs) excluding zero, are listed in Tables 5 and 6. For the ratio traits, only the significant correlations with absolute values > 0.2 are listed in Table 6. All estimates for the genetic correlations between behavioral and carcass traits are listed in [Additional file 1, Tables S8 and S9] and illustrated in [Additional file 2, Fig. S5]. Notably, because inference was performed within a Bayesian framework and significance was determined by whether the 95% HDI excluded zero, the multiple testing issues associated with frequentist hypothesis testing were avoided. Of the 252 combinations between behavioral non-ratio and carcass traits, 20 combinations exhibited significant genetic correlations, whereas among the 756 combinations between behavioral ratio traits and carcass traits, 62 exhibited significant genetic correlations. Of these 62 ratio traits, the component (i.e., numerator and denominator) behavioral traits were distributed as follows: feeding (33), moving (23), lying (18), standing (7), ruminating while lying (20), ruminating while standing (7), and ruminating (16). This unbalanced tendency suggests that the ratio traits that exhibited significant genetic correlations were not detected randomly and thus have biological meaning.

**Table 5:** Significant genetic correlations between carcass and behavioral non-ratio traits^a^

Carcass trait	Behavior trait		Genetic correlation
	Metric	Class	
Carcass weight	Daily frequency	Feeding	−0.277
	Longest interval	Ruminating	−0.180
	Daily total duration	Ruminating while lying	0.163
	Daily total duration	Ruminating	0.172
	Average interval	Feeding	0.258
Rib eye area	Daily frequency	Ruminating	−0.154
	Average interval	Moving	0.183
Subcutaneous fat thickness	Longest interval	Ruminating while lying	−0.209
	Longest duration	Feeding	−0.208
	Daily total duration	Feeding	−0.188
	Longest interval	Standing	−0.167
	Daily frequency	Lying	0.170
	Daily frequency	Standing	0.193
Yield rate	Daily frequency	Ruminating	−0.224
	Longest interval	Feeding	−0.200
	Average interval	Ruminating while standing	0.144
	Average interval	Ruminating	0.154
	Average interval	Ruminating while lying	0.172
BMS	Daily frequency	Moving	−0.153
	Average duration	Ruminating while lying	0.160

^a^Complete results are listed in [Additional file 1, Table S8] and graphically illustrated in [Additional file 2, Fig. S5]. The 95% highest density intervals are also listed in [Additional file 1, Table S8]

**Table 6:** Significant genetic correlations with absolute values > 0.2 between carcass and behavioral ratio traits^a^

Carcass trait	Behavior trait		Genetic correlation
	Metric	Ratio	
Carcass weight	Daily frequency	Feeding/Ruminating	−0.270
	Daily frequency	Feeding/Ruminating while lying	−0.241
	Daily total duration	Moving/Ruminating	−0.208
	Daily frequency	Moving/Ruminating while lying	-0.207
	Average interval	Moving/Ruminating while lying	0.209
	Longest interval	Moving/Ruminating while lying	0.210
	Longest interval	Moving/Ruminating	0.213
	Average interval	Moving/Ruminating	0.214
	Average interval	Feeding/Ruminating while lying	0.248
	Average interval	Feeding/Ruminating	0.250
Rib eye area	Daily total duration	Moving/Lying	−0.212
	Average duration	Lying/Standing	0.277
	Longest duration	Lying/Standing	0.288
Subcutaneous fat thickness	Longest duration	Feeding/Ruminating while lying	−0.222
	Longest duration	Feeding/Ruminating	−0.220
	Longest interval	Moving/Lying	0.203
	Longest interval	Moving/Ruminating while lying	0.217
	Longest interval	Feeding/Ruminating	0.229
	Longest interval	Feeding/Lying	0.234
	Longest interval	Feeding/Ruminating while lying	0.269
Yield rate	Longest interval	Feeding/Ruminating while lying	−0.277
	Longest interval	Feeding/Ruminating	−0.232
	Average interval	Feeding/Ruminating	−0.224
	Longest duration	Lying/Standing	0.228
	Average duration	Lying/Standing	0.232
Beef marbling score	Average duration	Ruminating while lying/Ruminating	0.201

^a^Complete results are listed in [Additional file 1, Table S9] and graphically illustrated in [Additional file 2, Fig. S5]. The 95% highest density intervals are also listed in [Additional file 1, Table S9]

The genetic correlation estimates between the behavioral ratio and carcass traits may be affected by the genetic correlations between the component traits of the ratio traits. Therefore, genetic correlations between the component traits were compared between the ratio traits that exhibited significant genetic correlations with carcass traits and those that did not. The average genetic correlations were 0.29 (0.21) and 0.36 (0.26), respectively, suggesting no systematic bias in genetic correlations between the component traits and the significant and insignificant ratio traits.

CW showed significant genetic correlations with 30 behavioral traits [Additional file 1, Tables S8 and S9]. Daily frequency of feeding and feeding-related ratios were negatively correlated with CW (Tables 5 and 6). Daily total duration and daily frequency of moving-related ratios were also negatively correlated (Table 6). In these ratios, the denominators were ruminating or ruminating while lying. In contrast, the average intervals of feeding and feeding-related ratios were positively correlated with CW, as were the average and longest intervals of moving-related ratios (Tables 5 and 6). Again, the denominators were ruminating or ruminating while lying. Taken together, these results suggest that animals that feed less frequently and for shorter durations but ruminate more frequently and for longer durations tend to have larger CW. The results also suggest that animals that move less frequently and for shorter durations compared with ruminating tend to have larger CW. As suggested by Krpálková et al. [23], the ratio between rumination and activity may be related to feed efficiency. Thus, our results suggest that animals ruminating more frequently and longer than moving have higher feed efficiency, resulting in larger CW.

REA was genetically correlated with seven behavioral traits [Additional file 1, Tables S8 and S9]. Strong correlations were observed for ratio traits (Table 6). The ratio of daily total duration of moving to that of lying was negatively correlated with REA (Table 6). On the contrary, the ratios of longest and average durations of lying to those of standing were positively correlated. These results suggest that animals spending more time lying with respect to moving or standing have higher REA. These results are somewhat unexpected, as muscle development is generally enhanced by increased physical activity. Therefore, other mechanisms may contribute to the development of REA.

RT was not significantly correlated with any behavioral traits. SFT showed significant genetic correlations with 21 behavioral traits [Additional file 1, Tables S8 and S9]. Longest durations of feeding and feeding-related ratios were negatively correlated with SFT (Tables 5 and 6). In these ratios, the denominators were ruminating or ruminating while lying. In contrast, the longest intervals of feeding- and moving-related traits were positively correlated, again with ruminating or ruminating while lying as the denominators (Table 6). Taken together, these results suggest that animals who spent more time feeding than ruminating have thinner SFT. The shorter duration of rumination relative to feeding may disrupt nutrient utilization, resulting in thinner SFT.

Yield rate was significantly genetically correlated with 19 behavioral traits [Additional file 1, Tables S8 and S9]. Longest and average intervals of feeding or feeding-related ratios were negatively correlated (Tables 5 and 6). These ratios used ruminating or ruminating while lying as the denominators. Daily frequency of ruminating was also negatively correlated (Table 5). In contrast, the ratios of longest and average durations of lying to those of standing were positively correlated (Table 6). These results suggest that higher yield rates are associated with a behavioral pattern characterized by more frequent and longer feeding relative to rumination, as well as longer lying durations relative to standing durations. In terms of feeding and rumination behavior, this pattern was opposite to that observed for CW, where animals that fed less frequently relative to their rumination activity exhibited higher phenotypic values. These results suggest that animals with higher feed efficiency are not necessarily associated with higher yield rates.

BMS significantly correlated with five behavioral traits [Additional file 1, Tables S8 and S9]. The ratio of the average duration of ruminating while lying to ruminating was positively correlated with BMS (Table 6). Average duration of ruminating while lying was also positively correlated with BMS (Table 5). In contrast, negative correlations were observed for the daily frequency of moving (Table 5). These results suggest that animals that ruminate lying for longer periods and move less frequently tend to have a higher BMS.

Several studies have reported genetic correlations between feeding behavior and carcass traits in beef cattle [14–16]. Among these studies, Chen et al. [14] and Nkrumah et al. [16] reported correlations with CW, Longissimus muscle area, and carcass marbling score, of which the latter two traits correspond to REA and BMS in our study. Kelly et al. [15] reported correlations with CW. The reported genetic correlations of daily feeding duration (daily total duration of feeding) with CW were 0.31 [16], 0.08 and 0.39 (Angus and Charolais, respectively) [14], 0.55 [15], and 0.08 in our study [Additional file 1, Table S8]. The genetic correlations of feeding frequency (daily frequency of feeding) with CW were 0.33 [16], 0.06 and 0.52 (Angus and Charolais, respectively) [14], 0.2 [15], and 0.277 in our study (Table 5). Apparently, no consistent tendencies were observed among the studies. Similarly, genetic correlations with other carcass traits (REA and BMS) in [14, 16] also showed inconsistent tendencies. These inconsistencies would arise partially due to the difficulty in estimating genetic correlations, reflected in the typically large standard errors observed: 0.2–0.3 in previous studies and 95% HDIs of 0.23–0.48 in our study [Additional file 1, Tables S8 and S9]. Genetic correlation estimates may also be sensitive to the estimation approach employed. In the present study, phenotypic values for behavioral traits were adjusted to obtain one-to-one corresponding phenotypes (Methods). Although this adjustment was necessary, the variance component estimates derived from bi-trait analysis 2 were not always consistent with those obtained from the corresponding single-trait models [Additional file 1, Table S10]. Alternatively, the relationship between behavior and carcass traits may be non-linear (e.g., concave downward), which could contribute to instability in the genetic correlation estimates. Thus, further investigation is needed to better understand these genetic relationships. No previous reports were found regarding genetic correlations between other behavioral classes/metrics and carcass traits.

As described, ratio-based traits showed heritability and repeatability comparable with non-ratio traits. In addition, ratio-based traits were often genetically correlated with carcass traits, and their estimated genetic correlations provided finer insights into the relationships between behavioral and carcass traits than those obtained from non-ratio traits. Although this is the first attempt to analyze ratios between behaviors as quantitative traits, the results support including ratio-based traits in the analyses of behavioral traits to better understand the genetic mechanisms underlying cattle behavior.

### Genomic prediction accuracy

**Fig. 4:**
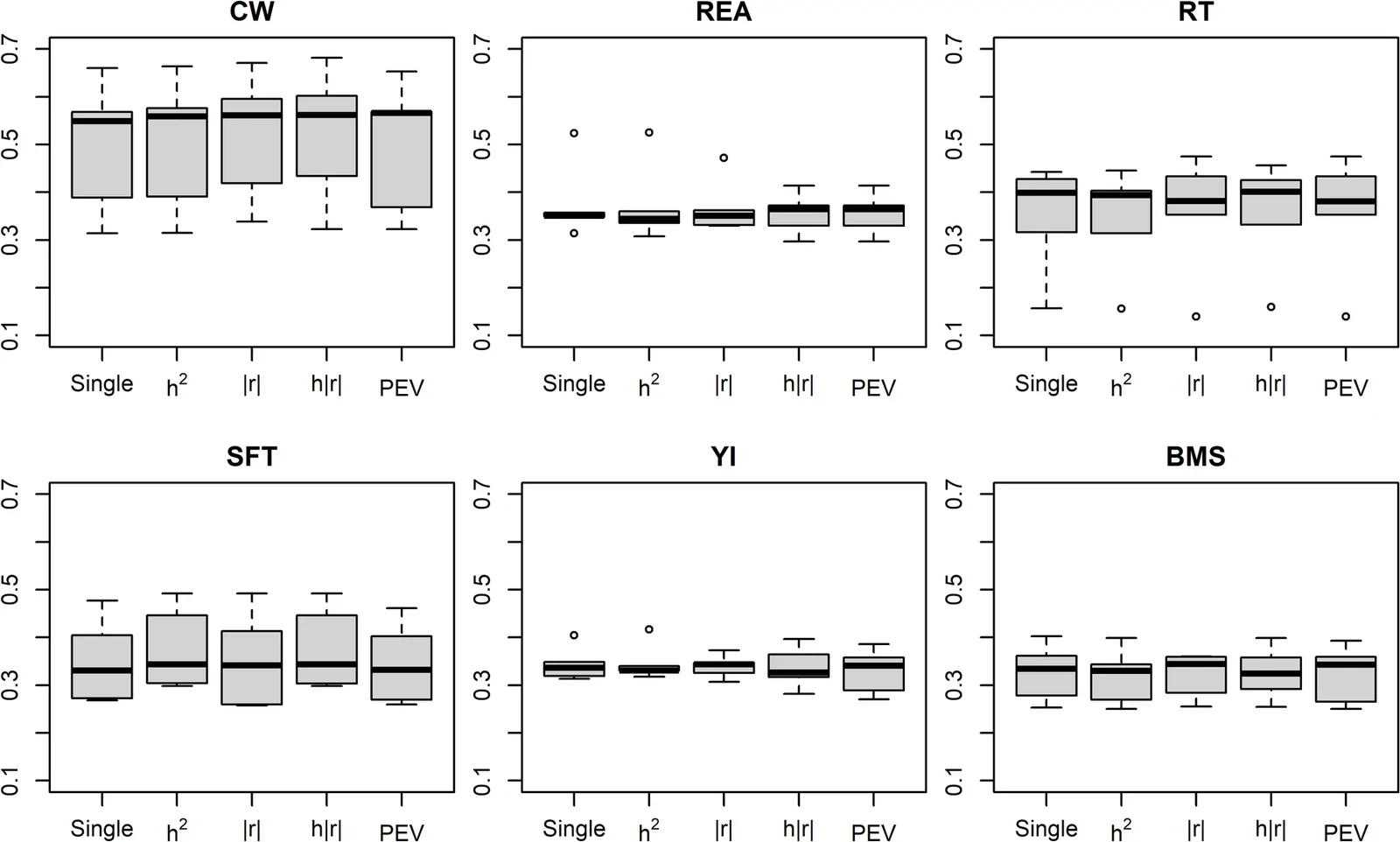
Prediction accuracy in cross-validation. Prediction accuracy obtained from single- and bi-trait models in cross-validation. The y-axis indicates the Pearson correlation coefficient between predicted additive genetic effects and observed phenotypes adjusted for fixed effects. Single: single-trait model for carcass traits, h^2^: bi-trait model implementing the secondary trait selected based on heritability ($\:{h}^{2}$), $\:\left|\mathrm{r}\right|$: bi-trait model implementing the secondary trait selected based on the absolute genetic correlation ($\:\left|r\right|$), $\:\mathrm{h}\left|\mathrm{r}\right|$:bi-trait model implementing the secondary trait selected based on the product of * h* and $\:\left|r\right|$, PEV: bi-trait model implementing the secondary trait selected based on prediction error variance, CW: carcass weight, REA: rib eye area, RT: rib thickness, SFT: subcutaneous fat thickness, YI: yield rate, and BMS: beef marbling score.

Cross-validation results are shown in Fig. 4. The selected behavioral traits used as secondary traits are presented in [Additional file 1, Table S11]. For each carcass trait, bi-trait models implementing behavioral traits as secondary traits showed prediction accuracies equivalent to those of single-trait models implementing only the carcass trait (Fig. 4). Furthermore, the rules to select secondary traits had little influence on accuracy. However, these results are reasonable considering the low heritability of behavioral traits and their low genetic correlations with carcass traits. Genomic prediction using bi-trait models is beneficial for focal traits with lower heritability, and the benefit increases when the correlated trait has a higher genetic correlation [20, 37]. Because carcass traits in this study had heritabilities of 0.44–0.72, which were higher than those of behavioral traits (Tables 3 and 4, and Additional file 1, Table S3), combining behavioral and carcass traits in bi-trait models does not substantially improve prediction accuracy for carcass traits. Thus, behavioral traits are likely not helpful for selecting bull candidates for carcass traits.

The generally low heritability estimates observed for behavioral traits may be attributable, at least in part, to phenotyping accuracy associated with the U-motion system and to the use of simplified statistical models Eqs. (1) and (3). Regarding the former, the choice of wearable device or measurement method may influence residual variance through differences in measurement error. Regarding the latter, the behaviors of cattle in the pen or paddock will be affected by various factors, such as pen area, pen density, and social interactions; however, these factors were not considered in the current models, which may result in increased residual variances. Thus, improving statistical models may be needed to use behavioral traits for breeding purposes. Nevertheless, when the current measurement devices and statistical models are used, behavioral traits may still be beneficial for predicting breeding values of other traits with low heritability; for example, reproductive traits, such as sperm volume and sperm mortality (the heritability was 0.175 and 0.081, respectively, in the Japanese Black cattle [38]). Genetic correlations between behavioral and reproductive traits will be of interest for future studies.

## Conclusions

In this study, heritability estimates for behavioral traits were generally low, ranging from 0.02 to 0.37 and accounting for 22.7%–67.5% of the repeatability. Genetic correlations between behavioral and carcass traits were also low in magnitude (− 0.28 to 0.29); however, significant correlations were detected for all carcass traits except rib thickness, and these genetic correlations showed interpretable patterns. Ratio-based behavioral traits were heritable and often genetically correlated with carcass traits, providing additional interpretability. Incorporating behavioral traits as secondary traits in multi-trait mixed-effects models did not improve the accuracy of genomic predictions for carcass traits, likely due to the relatively low heritabilities and weak genetic correlations of behavioral traits. Despite the generally low parameter estimates, the results offer novel insights into the genetic relationships between behavioral and carcass traits in beef cattle. These findings provide foundational information that may support the effective use of behavioral traits in breeding and management strategies for beef cattle.

## Supplementary Information

Below is the link to the electronic supplementary material.

Supplementary Material 1

Supplementary Material 2

## Data Availability

The phenotypes and genomic relationship matrices are provided upon request.
