# Supplementary material for: Heritability of behavioral traits and their genetic correlations with carcass traits in beef cattle
Source: Genet Sel Evol. 2026 Aug 24;58:61. doi: 10.1186/s12711-026-01082-5 (PMC13501594; doi:10.1186/s12711-026-01082-5)
Supplement: Supplementary file 1 [file 12711_2026_1082_MOESM2_ESM.pdf]

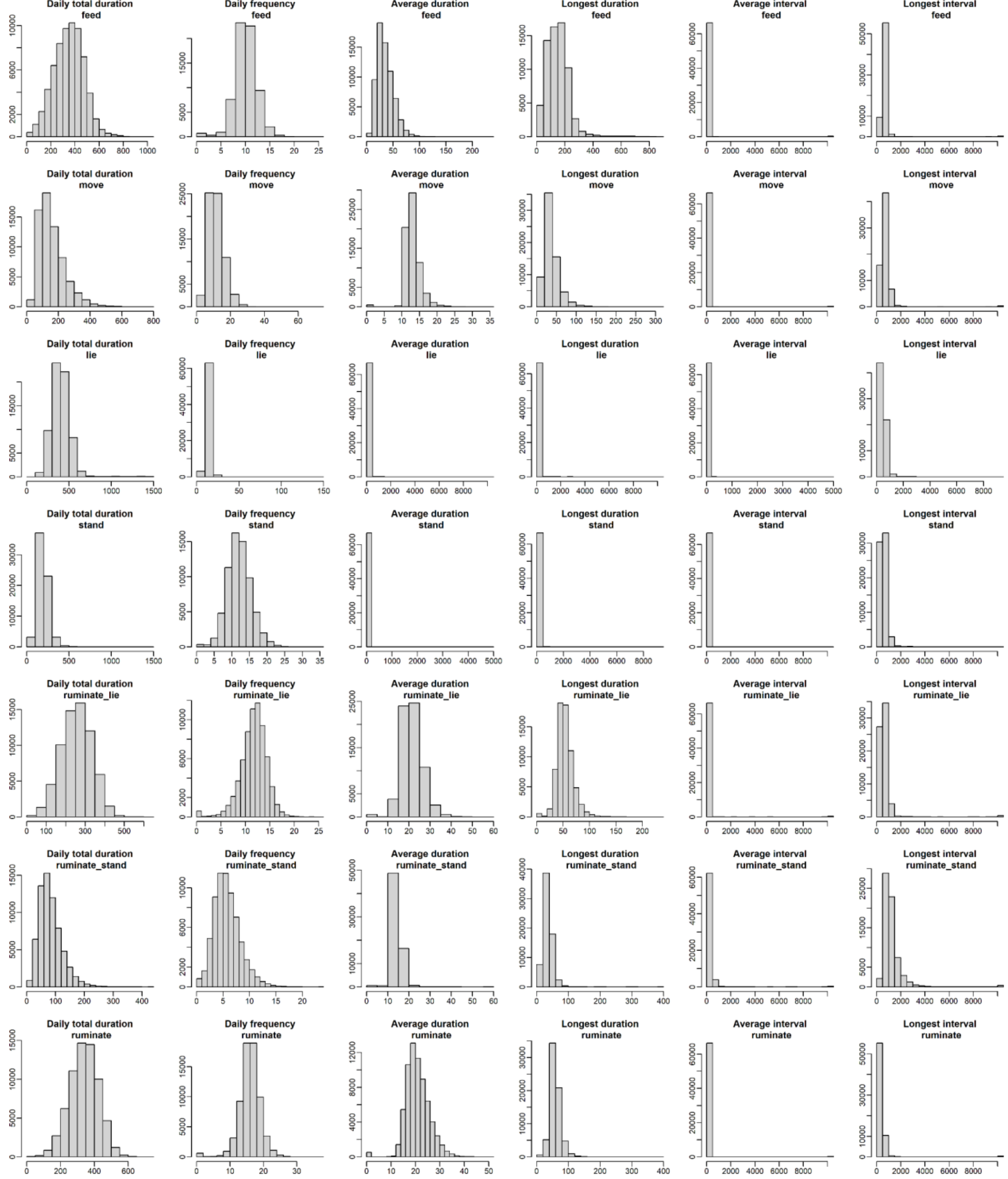

Figure S1 Distributions of phenotypic values of behavioral traits.

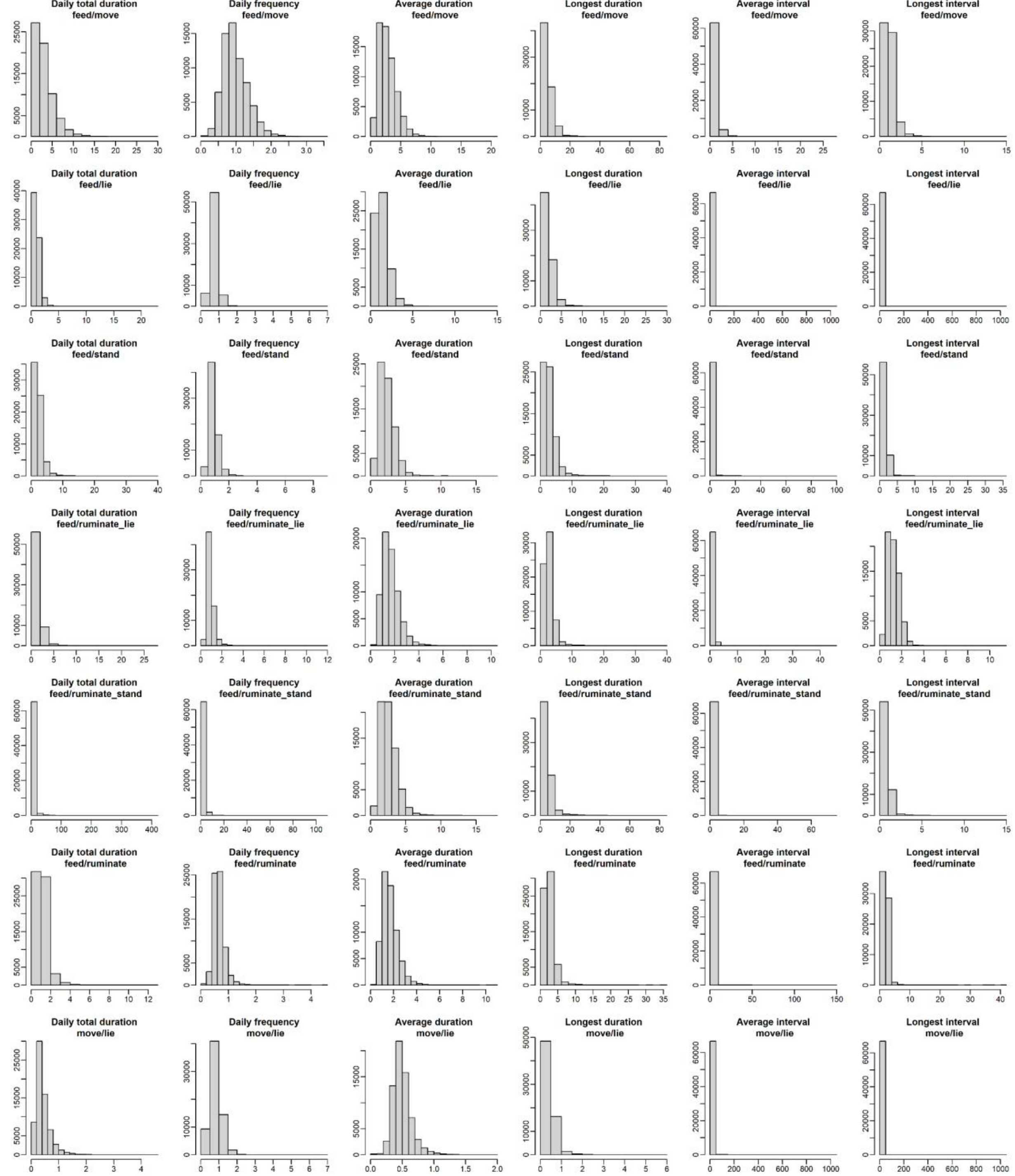

Figure S2 Distributions of phenotypic values of behavioral ratio traits.

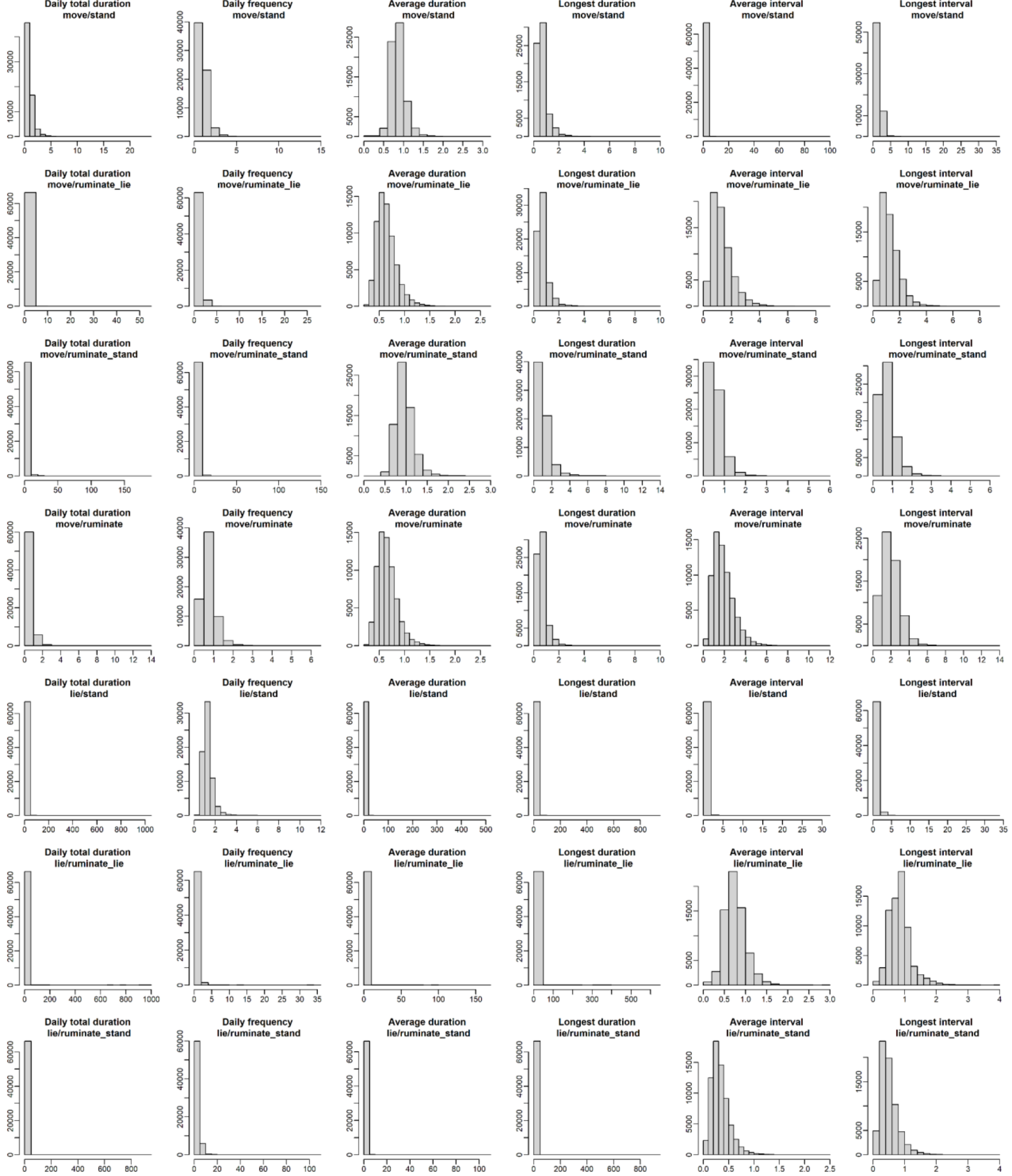

Figure S2 continued

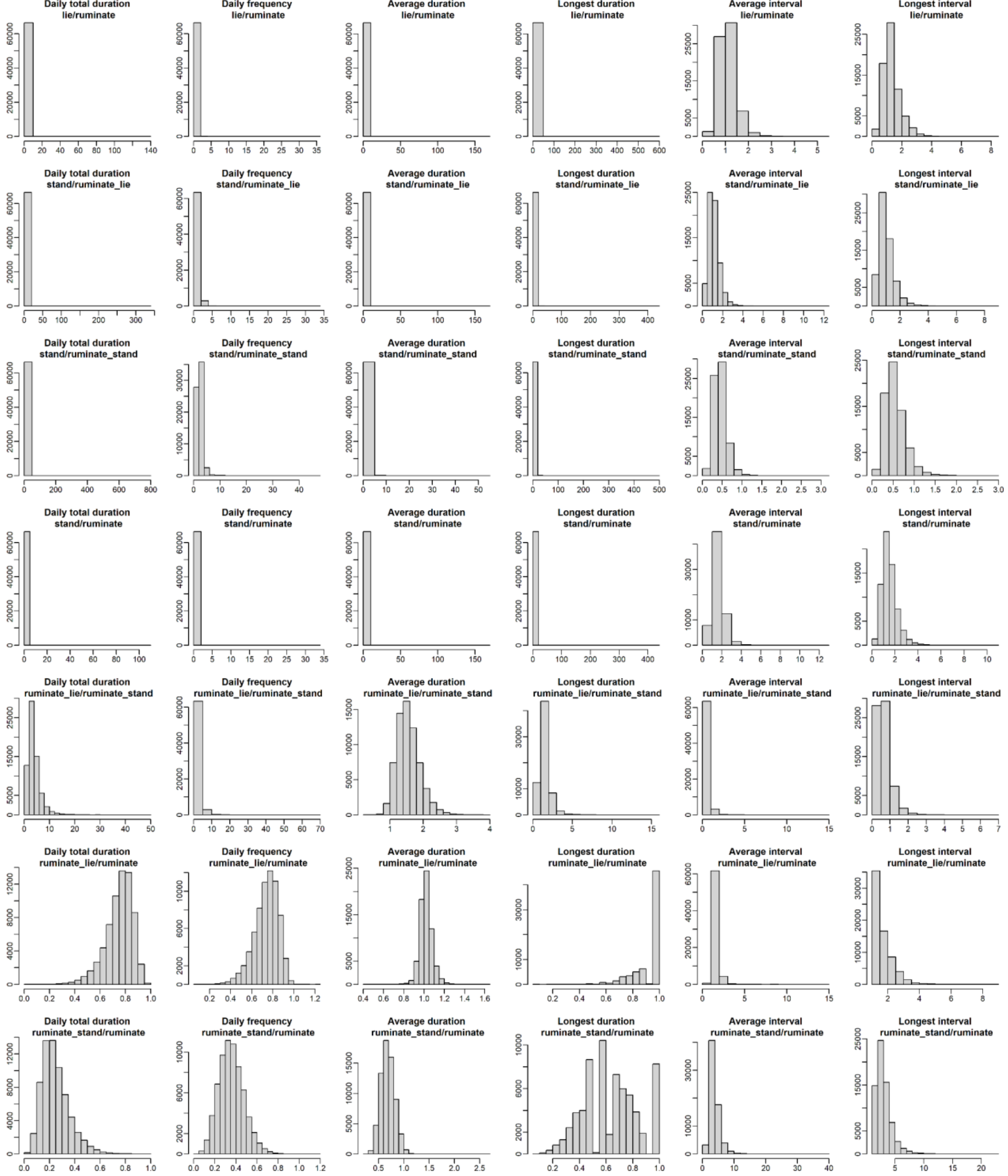

Figure S2 continued

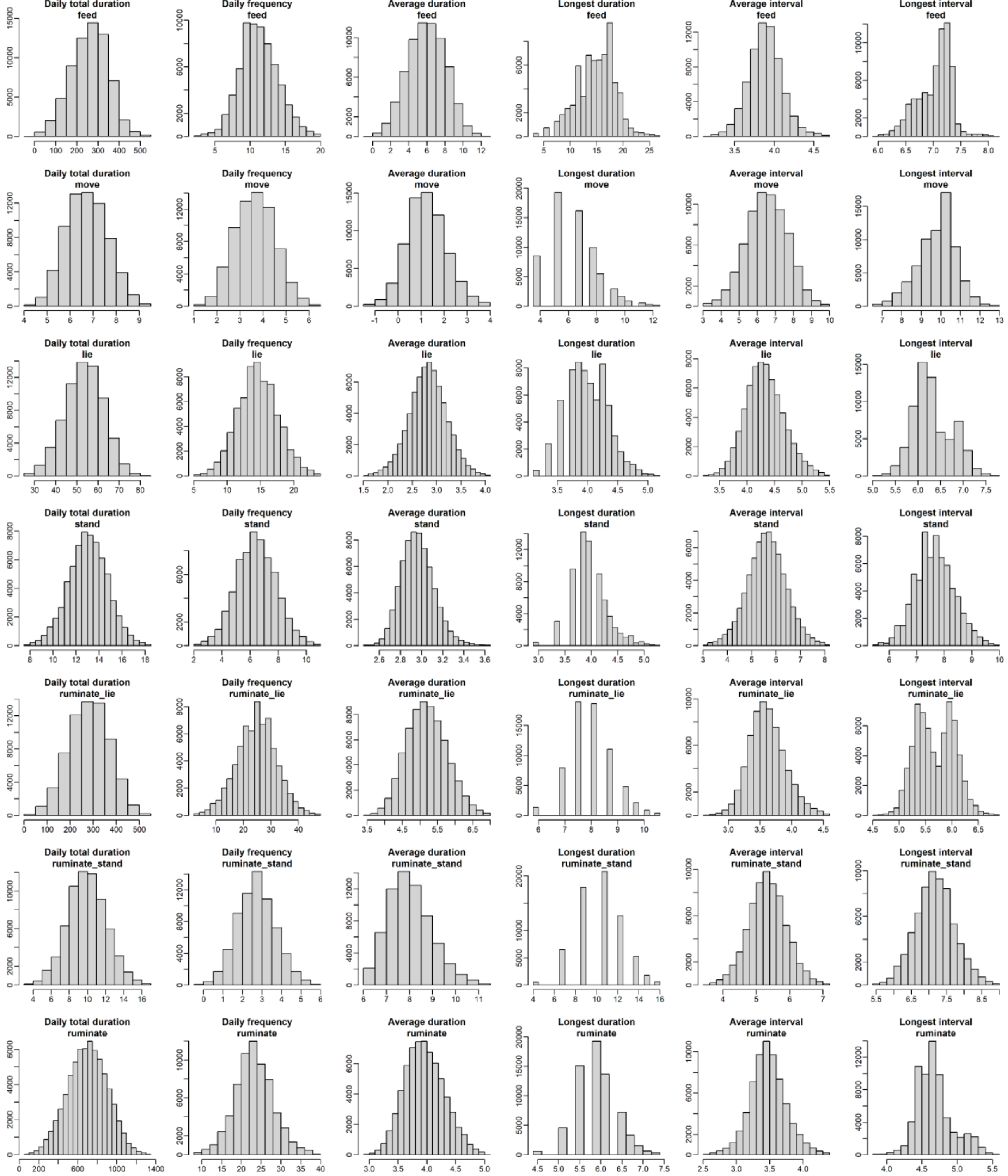

Figure S3 Distributions of transformed phenotypic values of behavioral traits.

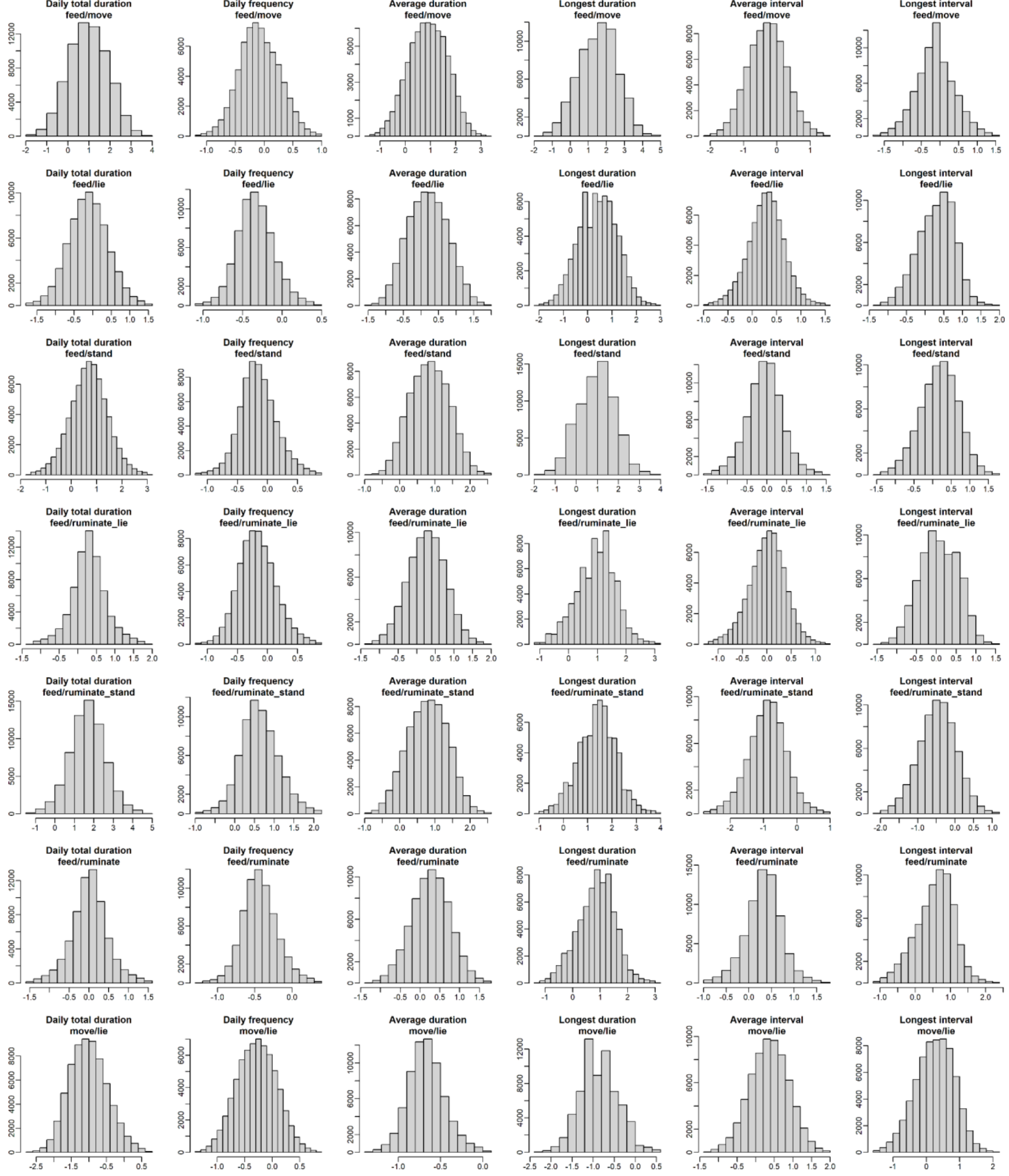

Figure S4 Distributions of transformed phenotypic values of behavioral ratio traits.

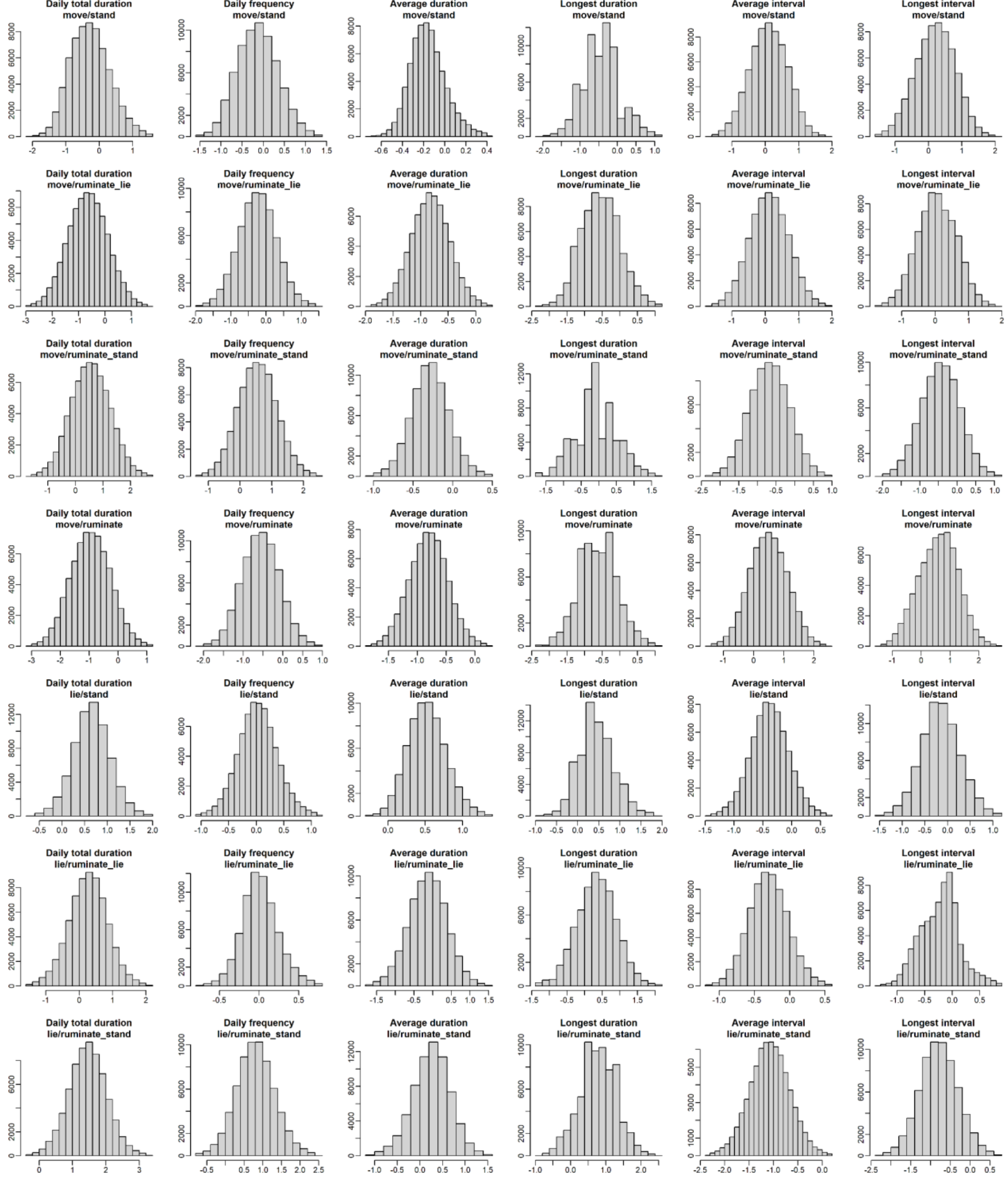

Figure S4 continued

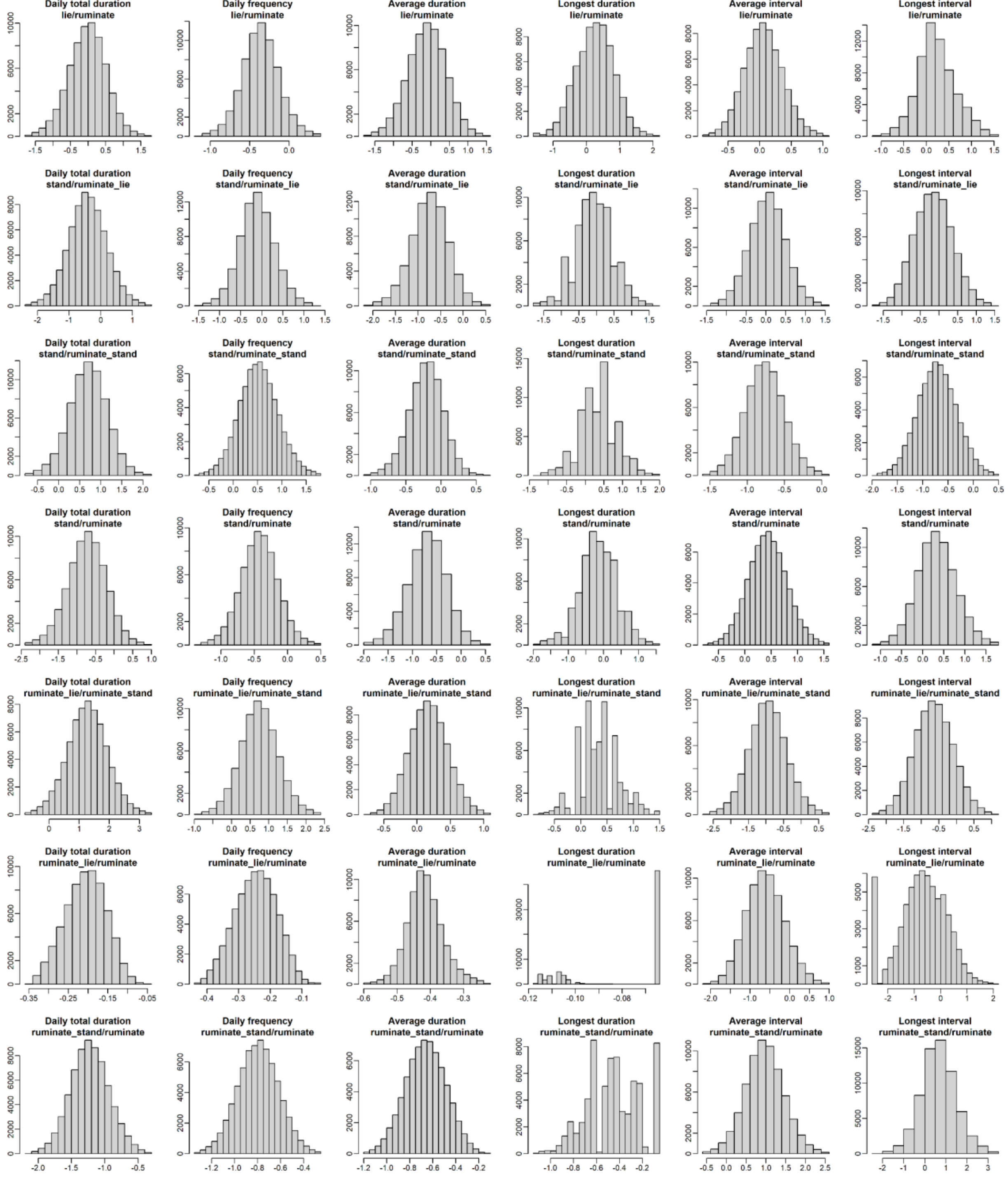

Figure S4 continued

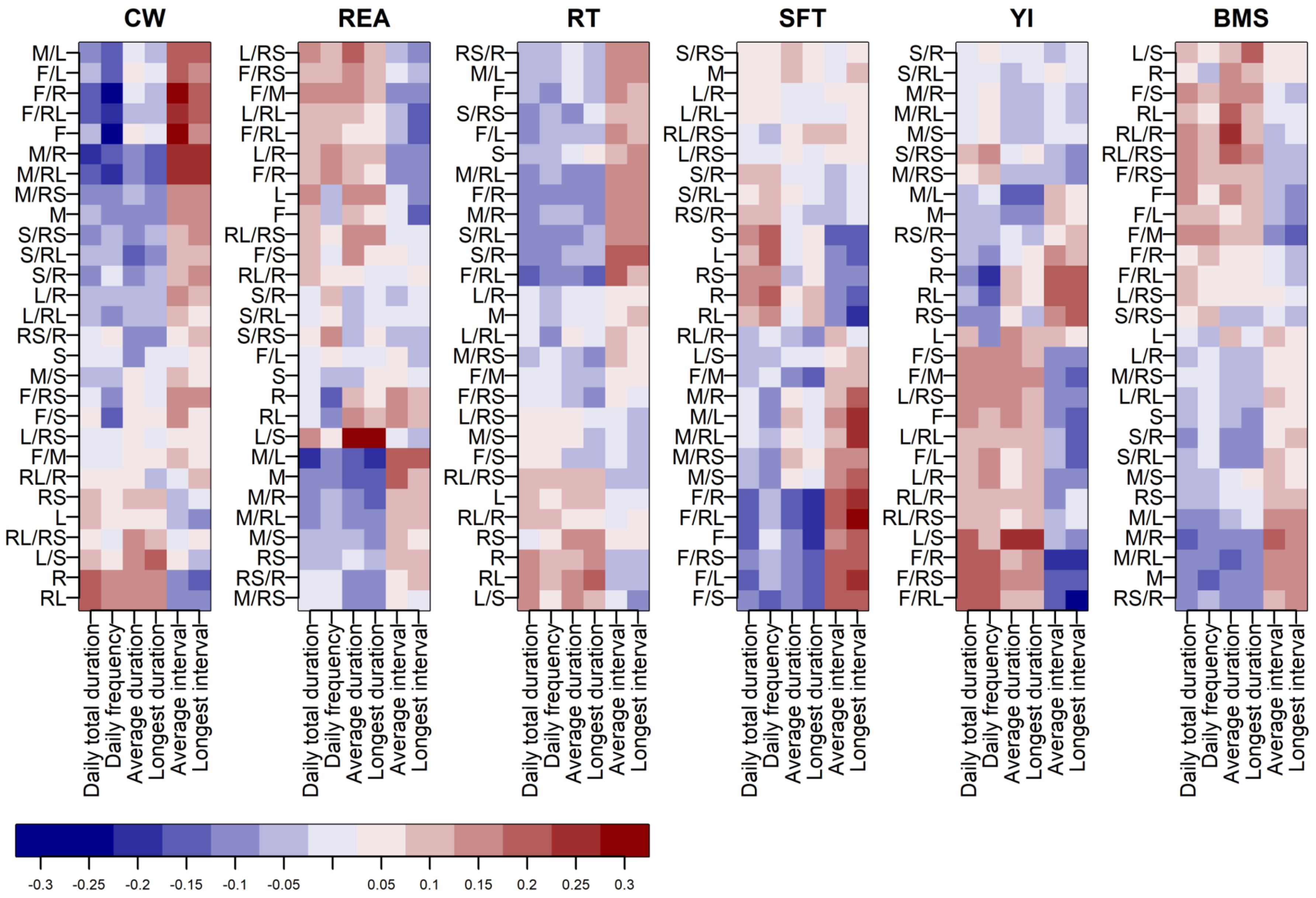

Figure S5 Genetic correlations between carcass and behavioral traits. Estimates are also presented in [Additional file 1, Table S5] and Table 2. CW: carcass weight, REA: rib eye area, RT: rib thickness, SFT: subcutaneous fat thickness, YI: yield rate, BMS: beef marbling score, F: feeding, M: moving, L: lying, S: standing, RL: ruminating while lying, and RS: ruminating while standing.
